# What Are the Functional Roles of Piwi Proteins and piRNAs in Insects?

**DOI:** 10.3390/insects14020187

**Published:** 2023-02-14

**Authors:** Dulce Santos, Min Feng, Anna Kolliopoulou, Clauvis N. T. Taning, Jingchen Sun, Luc Swevers

**Affiliations:** 1 Research Group of Molecular Developmental Physiology and Signal Transduction, Division of Animal Physiology and Neurobiology, Department of Biology, KU Leuven, Naamsestraat 59, 3000 Leuven, Belgium; 2 Guangdong Provincial Key Laboratory of Agro-Animal Genomics and Molecular Breeding, College of Animal Science, South China Agricultural University, Guangzhou 510642, China; 3 Insect Molecular Genetics and Biotechnology, Institute of Biosciences & Applications, National Centre for Scientific Research “Demokritos”, Aghia Paraskevi, 15341 Athens, Greece; 4 Department of Plants and Crops, Faculty of Bioscience Engineering, Ghent University, 9000 Ghent, Belgium

**Keywords:** RNAi, Piwi proteins, piRNAs, transposon control, stem cell function, antiviral defense, *Drosophila melanogaster*, *Aedes* mosquitoes, *Bombyx mori*

## Abstract

**Simple Summary:**

Three small RNA pathways, representing miRNAs, siRNAs and piRNAs, exist in insects. Of the three pathways, piRNAs and Piwi proteins appear to have the most complex biogenesis and functional roles. Significant progress has recently occurred in the understanding of the piRNA pathway in three major research areas, i.e., oogenesis and spermatogenesis in the fruitfly, antiviral response in mosquitoes and piRNA biogenesis in silkworm-derived BmN4 cells. Thus, it is timely to provide an overview of the knowledge accumulated so far. Studies in other insects are lagging behind but are highly anticipated since piRNAs and Piwi proteins are abundantly expressed not only in the germline but also in the somatic tissues. Due to the diversification of *Piwi* genes among insects, novel functions of piRNAs and Piwi proteins can be expected in the different species. This comprehensive review aimed to provide important background information about the current knowledge on piRNAs and Piwi proteins in insects that can function as a resource for future research.

**Abstract:**

Research on Piwi proteins and piRNAs in insects has focused on three experimental models: oogenesis and spermatogenesis in *Drosophila melanogaster*, the antiviral response in *Aedes* mosquitoes and the molecular analysis of primary and secondary piRNA biogenesis in *Bombyx mori*-derived BmN4 cells. Significant unique and complementary information has been acquired and has led to a greater appreciation of the complexity of piRNA biogenesis and Piwi protein function. Studies performed in other insect species are emerging and promise to add to the current state of the art on the roles of piRNAs and Piwi proteins. Although the primary role of the piRNA pathway is genome defense against transposons, particularly in the germline, recent findings also indicate an expansion of its functions. In this review, an extensive overview is presented of the knowledge of the piRNA pathway that so far has accumulated in insects. Following a presentation of the three major models, data from other insects were also discussed. Finally, the mechanisms for the expansion of the function of the piRNA pathway from transposon control to gene regulation were considered.

## 1. Introduction

In insects, three distinct small RNA silencing pathways are recognized: small interfering RNA (siRNA), micro-RNA (miRNA) and Piwi-interacting RNA (piRNA) [1]. Both siRNA and miRNA pathways are triggered by double-stranded RNA (dsRNA) which are processed by Dicer enzymes to small RNAs. During miRNA biogenesis, primary miRNAs correspond to endogenous transcripts with hairpin-like structures that are processed to precursor miRNAs by Drosha in the nucleus and subsequently to mature miRNAs by Dicer-1 in the cytoplasm [2]. The mature miRNAs become associated with RNAi-induced silencing complexes (RISCs) that contain Argonaute-1 (Ago1) as central factor and their main function consists of the interaction with the 3′-UTR of target mRNAs to induce their translational repression and/or degradation [3]. As regulators of gene expression at the posttranscriptional level, miRNAs play major roles in the control of cellular homeostasis and the adjustment of organism development [3].

In contrast to miRNAs, siRNAs are dedicated to the defense of the cells against parasitic elements. To achieve this aim, both the biogenesis and effector functions of the siRNAs are separated from the miRNAs by the employment of distinct Dicer and Argonaute molecules (Dicer-2 and Ago2, respectively) [4]. Triggers for the activation of the siRNA pathway are long dsRNA molecules that typically accumulate in the cell during RNA virus replication (exo-siRNAs) or from transcription of repetitive sequences in the genome (endo-siRNAs) [4]. The siRNA pathway targets transcripts from parasitic elements for degradation and is considered the main mechanism that protects against viral infection in insects [5,6].

The piRNA pathway has several conspicuous features that makes it clearly distinct from both miRNA and siRNA pathways. First, the trigger molecule of the piRNA pathway is not dsRNA but single-stranded RNA (ssRNA), and the generation of piRNAs is therefore independent of Dicer enzymes [7]. Instead, ssRNA precursors are processed by a set of interacting enzymes (of which also Piwi proteins) that result in the production of different types of piRNAs that are indicated as “initiator” (primary), “responder” (secondary) and “phased” (tertiary) [8]. As a result, piRNAs (27–32 nt) have a different (larger) size than miRNAs and siRNAs (19–22 nt) and are characterized by the enrichment of bases at specific positions [9]. A second difference is that the main effectors of this pathway belong to a separate subclass of Argonaute proteins called Piwi, distinct from the subclass AGO to which Ago1 and Ago2 belong. In this regard, another peculiar feature is that Piwi proteins have both initiator and effector functions. Most intriguingly, piRNAs often comprise the most abundant class of small RNAs in a tissue that map not only to transposable elements (indicating a function in genome defense) but also to cellular genes (indicating a function in gene regulation) [7,10]. The piRNA pathway, therefore, can have functions that overlap with both miRNAs and siRNAs.

Research on the piRNA pathway has until now been performed mainly in three insects: the fruitfly *Drosophila melanogaster*, the yellow fever mosquito *Aedes aegypti* and the silkworm, *Bombyx mori*. Their contributions to the understanding of the piRNA pathway are separate but also complementary and it is therefore worthwhile to integrate the findings from the three examples to obtain a global understanding of the complexity and the evolution of the piRNAs and the Piwi proteins. In the fruitfly, research on piRNAs has mainly focused on the gonads where it was revealed that the piRNA pathway in the somatic cells differed significantly from that in the germline cells [7]. In addition, significant differences were observed between testes and ovaries [11]. In the mosquito, expansion of the number of Piwi genes was observed and their expression in somatic cells was found to be associated with a role in antiviral defense [12,13]. The study of a silkworm cell line that expresses a functional piRNA amplification mechanism has greatly contributed to the understanding of piRNA biogenesis [14]. Furthermore, the study of chromosomal sex determination in the silkworm provided insights into the evolution of the piRNA pathway from transposon control to the regulation of physiological processes [15]. More recently, research on piRNAs and Piwi proteins has expanded to other insects, such as locusts, hemipterans, beetles and bees, that has already led to further insights in the potential complexity of the piRNA pathway.

**Figure 1:**
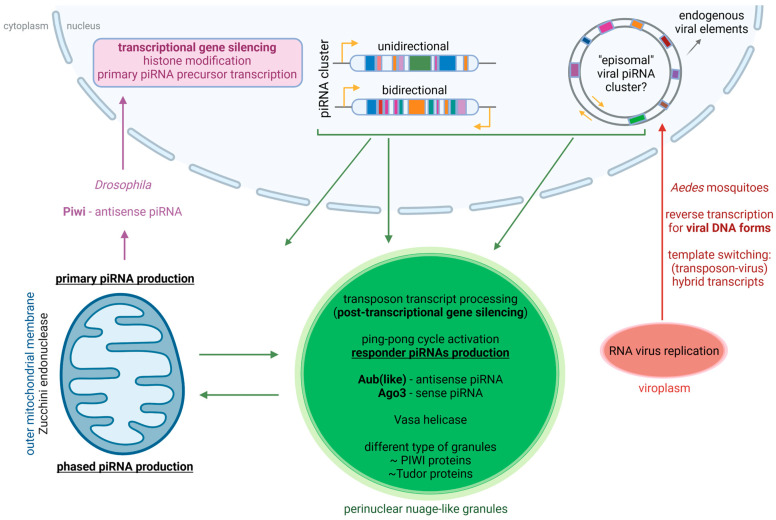
Overview of the piRNA pathway in insects as compiled from research (mainly) carried out in *Drosophila melanogaster*, *Aedes aegypti/albopictus* and *Bombyx mori*. The piRNA pathway is triggered by primary piRNA precursor transcripts that originate from genetic loci called piRNA clusters. The piRNA clusters typically contain the remnants of transposon sequences that are transcribed preferentially in antisense orientation. The primary transcripts are recruited to the outer mitochondrial membrane for processing into primary (initiator) piRNAs. In the perinuclear nuage-like granules, transposon transcripts are cleaved by piRNA-loaded Piwi proteins which results in the activation of the ping-pong cycle and the production of secondary (responder) piRNAs. Expansion of the diversity of piRNAs can also occur at the mitochondria through the process of phased (tertiary) piRNA production. The action of Aub-like and Ago3 Piwi proteins results in post-transcriptional silencing in the cytoplasm. In somatic ovarian cells of *Drosophila*, transcriptional gene silencing is observed by Piwi in the nuclei. The nuage-like granules correspond to (membraneless) biomolecular condensates that concentrate critical RNA and protein components by phase separation to increase the efficiency of biochemical processes in the piRNA pathway. Evidence exists for the existence of granules with specific composition that are dedicated to particular parts in the pathway and the silencing of specific groups of transcripts. In *Aedes* mosquitoes, the piRNA pathway has also evolved into an antiviral defense mechanism. The production of viral piRNAs (vpiRNAs) has been shown to require interaction with retrotransposons and the production of viral DNA forms. Eventually, viral DNA forms are hypothesized to become integrated in the cellular genome and function as piRNA clusters known as endogenous viral elements (EVEs). For more details on the biogenesis of piRNAs and the function of Piwi proteins, see the main text. This figure was created by BioRender.com.

This review has five main parts: in the first three parts, data from the three major models (fruitfly, mosquito, silkworm) are discussed in detail; the fourth part covers the progress in piRNA research in several other insects; finally, an overview is given that highlights the specific properties of the piRNAs and Piwi proteins encountered in the previous parts and that elaborates on the potential role of the piRNAs and Piwi proteins in the regulation of physiological and developmental processes. A schematic overview of the piRNA pathway (accumulated mainly from research in the fruitfly, mosquitoes and silkworm) is presented in Figure 1. The complex functions of piRNAs and Piwi proteins in different tissues of insects are also summarized in Table 1. An overview of the Piwi proteins in the different insects that are discussed in this review is provided in Supplementary Table S1.

## 2. Insights from Research on Piwi Proteins and piRNAs in *Drosophila*

### 2.1. Molecular Function of Argonaute Proteins of the Piwi Subfamily in Drosophila

Argonaute proteins of the Piwi subfamily are implicated in transposon control by an RNAi mechanism. Because of the evolutionary arms race with transposons, the piRNA pathway evolves rapidly and Piwi proteins have duplicated extensively across insects and arthropods [16]. In *Drosophila melanogaster*, three Piwi-subfamily members are present, i.e., Piwi, Aubergine (Aub) and Argonaute-3 (Ago3) that are preferentially expressed in the gonads [17].

#### 2.1.1. Molecular Functions of *Drosophila* Piwi Proteins in the Ovary

In the ovary, all three Piwi members are expressed in the germline cells that form the oocyte-nurse cell complexes in the egg chambers or follicles, while in the somatic cells of the follicular epithelium only Piwi is present [18].

A major part of the piRNAs in the ovary originates from genomic loci that consist of a large number of transposon sequences and are called “piRNA clusters” [7,19]. Long non-coding transcripts of the piRNA clusters become processed by endonucleases such as Zucchini and the Piwi proteins themselves to generate piRNAs of 25–29 nt [20]. However, different piRNA clusters and piRNAs are expressed in somatic and germline cells of the ovary: e.g., the uni-directionally transcribed *flamenco* cluster protects against long-terminal repeat retrotransposons in the soma while mainly dual-strand clusters are each associated with other families of transposable elements in the germline [17,21].

In somatic cells of the ovary, where only Piwi is expressed, piRNAs are generated by a “primary pathway” that does not employ a secondary amplification mechanism as observed in the germline cells [22] (see further below). Perinuclear cytoplasmic “spots”, known as Yb bodies, serve as centers for processing of piRNA precursors and piRNA-based RISC (piRISC) formation [23]. Piwi is a nuclear protein and functions in transcriptional gene silencing [24]. After their assembly at Yb bodies, piRISC complexes are transported to the nucleus and recruited to complementary nascent transcripts which results in heterochromatin formation following trimethylation of histone H3 at Lysine 9 at the targeted loci (typically containing transposon insertions) [9,25,26]. Sequence analysis revealed that the piRNAs bound to Piwi in the primary pathway are enriched at the 5′-terminus for Uracil (1U bias) which is accomplished by enrichment during Zucchini cleavage and preferential binding to the Piwi MID domain [27]. piRNA precursors bound to Piwi (as well as those bound to Aub and Ago3, see further below) at their 5′-end become trimmed at their free 3′-end by endonucleases followed by 2′-O-methylation of the 3′-termini, which is thought to increase stability [9,28].

In germline cells of the ovary, cytoplasmic Aub and Ago3 are co-expressed together with nuclear Piwi [7]. Processing of piRNA precursors and piRISC formation also occurs in nurse cell complexes at perinuclear organelles called “nuage” of which the assembly likely depends on Yb homologs that are expressed in the germline [29]. Because of the expression of Aub and Ago3, however, the primary piRNA pathway becomes linked to the amplification loop designated as “ping-pong” to produce secondary piRNAs that function in post-transcriptional gene silencing [9]. Aub complexes with primary piRNAs (with 1U bias and typically having antisense orientation; also called “initiator” piRNAs; [9]) cleave transposon targets, thereby creating the 5′-ends of the next generation of piRNAs (in sense orientation) that become loaded into Ago3 (as secondary or “responder” piRNAs; [9]). This arrangement results in an overlap of 10 nt between the Aub- and Ago3-associated piRNAs as well as an enrichment of A at position 10 (10A bias) in piRNAs of Ago3-complexes (ping-pong signature; [9]). After maturation, Ago3-piRNA complexes also engage in the cleavage of piRNA cluster transcripts to generate additional antisense piRNAs that can target the transposons (resulting in a more robust silencing mechanism in the germline). In addition, Piwi/Aub-piRNA precursor complexes (in germline and soma) can become involved in the phased processing of piRNA precursors following recruitment of Zucchini endonuclease and the helicase Armitage downstream along the transcripts to generate (tertiary) “trailer” piRNAs [9,20,30]. In this model, the ping-pong loop results in the amplification of the piRNA response while the production of trailer piRNAs results in an expansion of the region of piRNA formation which results in a more efficient silencing of the target [9,28].

Conform to its role in transposon silencing, Piwi mutants show greatly increased levels of the *gypsy* retrotransposon in the somatic cells of the ovary [9]. Similarly, Aub mutations de-repress telomeric *TART* retrotransposons and P-elements (which are DNA transposons) in the germline [31,32]. The long terminal-repeat (LTR) retrotransposon *diver* and the LINE-like elements *Het-A* and *I* rely both on Aub and Ago3 for silencing [33]. In the germline, post-transcriptional silencing of transposable elements by Aub and Ago3 (with ping-pong amplification mechanism) also creates a pool of piRNAs that are bound by Piwi for transcriptional silencing in the nucleus [34].

For transposon silencing, piRNA clusters could function as triggers by the production of antisense sequences of transposons embedded in long transcripts that are processed by endonucleases such as Zucchini and Piwi subfamily Argonaute proteins. It was therefore proposed that piRNA clusters could function as adaptive loci since random integration of active transposons would result in the silencing of related transposons in “*trans*” (“jumping-in cluster” model; [35,36]). However, a recent study indicated that while the bulk of piRNAs in the ovary are produced by the piRNA clusters, deletion of the major piRNA clusters did not result in widespread de-silencing of transposons [21]. Instead, piRNAs that originate from numerous recent transposon insertions throughout the genome may be more active in the (in *trans*) regulation of transposon activity. A mechanism for conversion of active transposon insertions into piRNA-producing loci could involve the recruitment of Rhino-Deadlock-Cutoff complexes (which regulate non-canonical transcription in heterochromatin environments) following targeting by nuclear piRISC complexes [37,38]. Furthermore, large piRNA clusters in Drosophila are considered evolutionary labile and the piRNAs that they produce may have lost their relevance for the control of transposable elements after a short period of benefit [21]. Large clusters of transposable elements that are converted to piRNA clusters typically are localized in pericentromeric heterochromatin at sites that are not easily removed by recombination which explains their relative persistence at the micro-evolutionary time scale.

In *Drosophila*, the telomerase gene has been lost and the telomeres of the chromosomes are maintained by transpositions realized by telomeric retrotransposons (*HeT-A*, *TART*, and *TAHRE* retroelements; [39]). In germline cells (nurse cells of the ovary), the telomeric regions form dual-strand piRNA clusters of which the derived piRNAs auto-regulate the maintenance of telomeric heterochromatin. In somatic cells (eye-pigment cells in the ommatidia), on the other hand, the telomeric retrotransposon arrays are active while the telomere-associated sequences are silenced independent of the piRNA pathway by polycomb group proteins [40].

RNAi-mediated knockdown of Piwi, Aub or Ago3 does not affect strongly oogenesis although follicles degenerate in Aub/Ago3 double mutants at the late stage [34,41]. However, specific knockdown of Aub and Ago3 in the female germline causes female sterility and disruption of embryonic axis formation [33,42]. Since the developmental defects caused by loss of Aub function are suppressed by mutants in the DNA damage-signaling pathway [43], female sterility is proposed to result mainly from DNA damage following mobilization of transposons and the subsequent activation of the double-stranded break repair pathway associated with the meiotic cell cycle checkpoint. Checkpoint activation results in the disruption of the microtubule network that is necessary for axis specification in the oocyte and embryo. In addition, Aub is involved in the transport of mRNAs and piRNAs from the nurse cells in the developing egg chambers and their localization in the oocyte (see further below).

#### 2.1.2. Molecular Functions of *Drosophila* Piwi Proteins in the Testis

Similarly, in the testis, Piwi is expressed mainly in the somatic cells (but is also required autonomously for germline stem cell maintenance, see below) while Aub and Ago3 are enriched in the germline cell lineage [44]. In the ovary, piRNAs constitute the most abundant class of small RNAs but this is not the case for the testis where miRNAs and endo-siRNAs constitute a larger fraction [11]. Furthermore, piRNAs in the testis are less associated with transposable elements than in the ovary and also have adapted to other transposon families [11]. Genes in the piRNA pathway are expressed much less in the testis compared with the ovary which correlates with the observation that piRNA-mediated silencing of transposable elements is also much weaker [45].

In the testis, Aub and Ago3 also localize in the nuage of the germline cells [46,47]. Conventional nuage organelles appear in all stages including germline stem cells, spermatogonia and spermatocytes. During spermatocyte growth and differentiation, a much larger structure, the piRNA nuage giant (piNG) body, with 50-fold larger volume, is observed [47]. The internal structure of the piNG body indicates compartmentalization of distinct piRNA-related processes as well as a possible crosstalk with the miRNA pathway.

In contrast to the ovary, the majority of piRNAs in the testis are produced in the germline by two loci, *Suppressor of stellate* (*Su(Ste)*) on the Y chromosome (~43%) and *AT-chX* on the X chromosome (~31%) [48]. *Su(Ste)* represents a piRNA cluster that functions in the silencing of the expression of the Stellate (Ste) protein (located on the X chromosome) that, in the absence of specific piRNAs from the Y chromosome, aggregates into crystals in spermatocytes [48]. Recognized as the cause for sterility of XO males, the Ste-Su(Ste) system was proposed to have evolved as a checkpoint for accurate Y chromosome segregation. The *AT-chX* locus contains repeats that have similarity to the gene *vasa*, which is essential for gametogenesis [49]. However, antisense piRNAs of *AT-chX* do not target *vasa* transcripts in *D. melanogaster* but are complementary to *vasa* of *D. mauritiana* expressed in testes of interspecific hybrids thereby causing male sterility [49]. Thus, in the testis, both piRNA loci *Su(Ste)* and *At-chX* are involved in the regulation of fertility and the mediation of genetic conflict in D. melanogaster [50].

### 2.2. Piwi Subfamily Proteins and the Regulation of Stem Cell Function in the Gonads in Drosophila

#### 2.2.1. The Gene *piwi* in *Drosophila*

The gene *piwi* (for *P-element induced wimpy testis*) was first identified during a screen for mutations that affect the self-renewal of germline stem cells in both female and male gonads of adult *Drosophila melanogaster* flies [51].

##### The Gene *piwi* and the Regulation of Stem Cell Function in the Ovary in *Drosophila*

While Piwi is expressed in the germline cells of the ovary, its major requirement for germline stem cell maintenance does not reside in the germline but in the (somatic) terminal filament and cap cells, which constitute the “niche” for germline stem cell maintenance in the germarium region of the ovarioles [52]. In addition, the escort cells, a subpopulation of the inner germarial sheath cells, function to insulate germline cystoblasts and cystocytes [53]. Piwi was also shown to be required in escort cells for promotion of germ cell differentiation [42].

By contrast, removal of Piwi function in the ovarian germline stem cells allows normal oogenesis to occur but results in the arrest of embryogenesis that is not rescuable by paternal wild-type Piwi [52] (see further below). However, germline-specific knockdown of Piwi also causes a modest decrease in germline stem cells, indicating its cell-autonomous requirement [54]. Germline-specific Piwi function is necessary for the proliferation and survival of primordial germ cells in the developing ovary during larval-pupal development, and the formation of germline stem cells in the ovaries of the adult flies [42].

Although Piwi is involved in transposon silencing, this function can be separated from its role in germline cell maintenance [54]. For germline cell renewal, Piwi is mainly required in the somatic niche cells for the production of paracrine factors (e.g., Decapentaplegic (Dpp)) that signal to the germline stem cells [55]. In contrast to the nuclear function of Piwi for (transcriptional) transposon silencing, germline cell renewal by the somatic niche cells can be maintained by Piwi proteins that have a deletion in their N-termini and remain localized in the cytoplasm. Additionally, in the absence of Zucchini, which encodes an endonuclease necessary for piRNA production, germline stem cell renewal is not disturbed. However, the Yb protein is also indispensable for germline cell maintenance and localization of Piwi to the Yb bodies of niche cells could therefore constitute an essential signal for germline stem cell maintenance [24]. Interestingly, Piwi can repress the expression of the transcription factor c-Fos, involved in cell differentiation and proliferation, in the somatic niche cells of the ovary by a posttranscriptional mechanism [56]. By an unknown mechanism, Piwi can target the 3′-UTR of *c-fos* mRNA for production of piRNAs which results in the de-stabilization of the transcript [56]. Repression of *c-fos* by Piwi is not only essential for somatic niche cell function but also for normal egg chamber development at later stages of oogenesis.

The nuclear function of Piwi may nevertheless be required for germline stem cell maintenance, albeit also according to a mechanism that does not require piRNAs. Piwi was shown to interact with the Polycomb repressive complex 2 (PRC2) in the nucleoplasm to sequester PRC2 from its targets in the chromatin and preventing lysine-27-tri-methylation of histone 3 (H3K27m3) [57]. As a consequence, transcription by RNA Polymerase II becomes modified at a genome-wide level which can be interpreted as a mechanism for epigenetic programming of germline cell function. This unique function of Piwi is suggested to be germline-specific and results in the promotion of expression of genes involved in germline function that are inhibited by PRC2 [57].

The function of Piwi to silence transposons can have a more direct effect on cellular function and stem cell maintenance. It was reported that the de-silencing of transposons in *piwi* and *flamenco* mutants results in a decrease in the expression of the Wnt ligand dWnt4 which acts in an autocrine manner in the escort cells to regulate the encapsulation and differentiation of the cystoblasts which are the immediate progeny of the germline stem cells [58]. The sensitivity of escort cells to transposon mobilization may be a defense mechanism against the production of *gypsy* retroviruses in the soma that can infect developing germline cells within the germarium and cause damage during follicle and egg development [59].

Another mechanism by which Piwi can regulate gene expression is by the spreading of repressive chromatin marks from transposon insertions into neighboring cellular genes [41]. Since genes that are adjacent to transposable elements show higher expression in ovaries than in other tissues, Piwi may also function to prevent the ectopic transcription of inappropriate genes that occurs concomitantly with the transcriptional silencing of mobile elements [41].

##### The Gene *piwi* and the Regulation of Stem Cell Function in the Testis in *Drosophila*

In the testis, knockdown of Piwi in the (somatic) hub cells, which are essential for germline stem cell maintenance [60], did not result in a disruption of stem cell maintenance [61]. The stromal non-dividing hub cells in the testis support two stem cell populations: the germline stem cells (which provides the lineage that will differentiate into spermatocytes) and the somatic cyst stem cells (which give rise to the somatic cyst cells surrounding the germline cysts) [53,60]. While Piwi expression appears highest in the hub cells of the testis, reduction of Piwi in the hub nevertheless does not disrupt both stem cell populations and spermatogenesis [61]. On the other hand, despite its low expression in germline cells, Piwi was reported to be required autonomously for both germline stem cell and somatic cyst stem cell maintenance while it is dispensable for progression at later developmental stages [61]. Using a Piwi protein that is defective for nuclear localization, it was also established that the nuclear function of Piwi in somatic cyst cells is essential for somatic and germ cell differentiation [61].

In the soma of the testis, piRNAs were identified that are antisense to *Fasciclin 3* (*Fas3*), which encodes a transmembrane cell adhesion molecule that normally is highly expressed in the hub cells. Knockdown of Piwi causes ectopic expression of *Fas3* in cyst cells which results in the disruption of germline cyst development [61]. Thus, Piwi and specific piRNAs function in the soma to regulate the interactions between the germline and cysts cells during early stages of germ cell differentiation.

In the ovary, a very similar mechanism was identified that involves the *gene traffic jam* (*tj*), which encodes a Maf/basic Leucine Zipper transcription factor that regulates the interaction between somatic and germline cells during gonad morphogenesis. While Tj protein functions as an activator of Piwi transcription, it was also observed that the 3′-UTR of *tj* mRNA is processed into (sense) piRNAs in somatic cells [62]. Processing of the 3′-UTR is predicted to affect the stability of *tj* mRNA and the resulting piRNAs were also proposed to act in trans and regulate the expression of other cellular genes such as *FasIII*. However, less than 3% of piRNAs derived from the 3′-UTR of *tj* have (partial) complementarity to the large intron of *FasIII* and the impact on *FasIII* expression remains to be addressed experimentally [10,63]. With respect to the specificity of selection of the 3′-UTR of *tj* mRNA for piRNA processing, a 100 nt *cis*-acting element was identified that interacts with Yb protein to initiate piRNA production [64].

#### 2.2.2. The Genes *aub* and *ago3* in *Drosophila*

Besides nuclear Piwi, cytoplasmic Aub and Ago3 were also shown to have a role in the regulation of stem cell function in the gonads.

##### The Genes *aub* and *ago3* and the Regulation of Stem Cell Function in the Ovary in *Drosophila*

While germline-specific knockdown of Aub does not affect early germline development and germline stem cell formation in the ovary of newly eclosed female flies [42], Aub nevertheless is essential for germline stem cell renewal in the germarium and differentiation of the germline lineage during oogenesis [65]. Mutants in Ago3, on the other hand, do not show a defect in germline stem cell renewal although germline stem cell differentiation was disrupted [66].

Prevention of checkpoint activation partially underlies the capacity of Aub to maintain germline stem cell renewal [65,66]. However, Aub also regulates germline stem cell maintenance according to a mechanism that is independent of the prevention of transposon activation and that involves the regulation of cellular mRNAs [66]. Aub interacts with the CCR4-NOT de-adenylation complex, which is recruited to the 5′-UTR and 3′-UTR of the mRNA of *Casitas B-cell lymphoma* (*Cbl*) via complementary piRNAs that are present in the ovary [66]. *Cbl* encodes an E3 ubiquitin ligase that inhibits the activity of tyrosine kinases and plays a role in the maintenance of hematopoietic stem cells in mammals. However, translational repression of *Cbl* mRNA by Aub/CCR4-NOT (which is essential for germline stem cell renewal in the ovary) does not involve the shortening of its poly(A) tail. Moreover, since Ago3 does not play a role, piRNAs are proposed to be generated by a “homotypic” Aub/Aub ping-pong mechanism [33].

It was also proposed that Aub may regulate germline stem cell renewal by mechanisms that do not involve piRNAs. The iCLIP (individual-nucleotide resolution Cross-Linking and ImmunoPrecipitation) technique was used to identify the interaction of Aub with many mRNAs in an in vitro culture system of germline stem cells, including its own mRNA and mRNAs that express self-renewal (regulators of bone morphogenetic protein (BMP) signaling, adhesion to the niche and chromatin remodeling) and differentiation factors (RNA-binding proteins involved in repressing of BMP signaling and promoting *Bag-of-marbles* (*Bam*) expression) [65]. By interacting with the 3′-UTR of mRNAs and factors of the translation initiation eIF4 complex and poly(A)-binding protein, Aub is proposed to modify gene expression by regulating translation initiation.

However, the role of piRNAs can not completely be excluded in all cases and may be uncovered by the utilization of other rules for identification of piRNA-mRNA interactions. For instance, *dunce* mRNA (encoding a cAMP-specific phosphodiesterase) was identified as another Aub target while loss of germline stem renewal was observed following the deletion of a piRNA-binding site in the 3′-UTR of *dunce* [65,66]. More research is required to identify the mechanism of recruitment of Aub to mRNAs that may involve binding motifs [65] or the use of piRNAs as intermediaries [66].

##### The Genes *aub* and *ago3* and the Regulation of Stem Cell Function in the Testis in *Drosophila*

Male flies that are mutant in Aub or Ago3 are sterile and the sterility is related to the accumulation of crystals of Stellate protein in the primary spermatocytes which is caused by a failure in the production of *Su(Ste)* piRNAs (see the previous Section 2.1) [33,67]. In contrast to the ovary, the number of germline stem cells at the tip of the testes is greatly reduced in Ago3 mutants [33], indicating its role in the maintenance of this stem cell population.

### 2.3. Functional Roles of Piwi Proteins during Germ Cell Differentiation in Drosophila

#### 2.3.1. Functional Roles of *Drosophila* Piwi Proteins during Germ Cell Differentiation in the Ovary

During oogenesis, maternal mRNAs are deposited in the oocyte that become translated during early embryogenesis. At the maternal-to-zygotic transition, these maternal mRNAs then become massively degraded [68]. Individual nucleotide UV crosslinking and immunoprecipitation (iCLIP) assays revealed that a large number of maternal mRNAs during early embryogenesis are bound by Aub through piRNA-mediated interactions [69,70]. The maternal mRNAs bound by Aub are enriched for components of the germ plasm (the cytoplasm located at the posterior pole of the embryo that directs germ cell specification), indicating a mechanism for selective degradation in the somatic parts of the embryo and enrichment in the pole plasm [71]. Interaction with maternal mRNAs guided by piRNAs occurs preferentially at the 3′-UTR and results in the recruitment of the CCR4-NOT de-adenylation complex together with the RNA-binding protein Smaug [72].

Maternal mRNAs are produced in the nurse cells during oogenesis and, similarly to transcripts from piRNA clusters and transposable elements, are proposed to be scanned by piRNAs in the nuage to form piRISC complexes [71]. However, while mRNAs of transposons are heavily targeted by perfect complementary piRNAs, regulation of maternal mRNAs is considered less severe because of more imperfect base pairing [69]. In the case of *nanos* mRNA, targeting piRNAs originate from the LTR retrotransposons *roo* and *412* which interact through incomplete base pairing by a miRNA-like mechanism [72]. Similarly, *dunce* maternal mRNAs are targeted by piRNAs of the non-LTR transposon *R1* [69]. These examples illustrate the adaptation of transposable elements to the regulation of developmental processes [71]. 

Besides its role in the degradation of transposon mRNAs in the germline and the elimination of maternal mRNAs in early embryos, Aub also appears as one of the core components of the pole plasm at the posterior pole of the oocyte that will determine primordial germ cell development [73,74]. Because of the association with piRNAs that represent transposable elements, Aub- and Piwi-RISC complexes in the germline cells constitute a protective mechanism against invading transposons from the paternal genome following fertilization [48]. Thus, the phenomenon of hybrid dysgenesis that occurs in crosses in which males carry transposons that females lack can be explained by the absence of maternally inherited piRNAs targeting the paternal transposons [75]. The piRNAs that are maternally transmitted also function as epigenetic signals for the activation of piRNA production in homologous regions of the genome in the progeny [76].

Aub-piRNA complexes are proposed to play an active role in the anchoring of maternal mRNAs to the germ plasm [70]. In this case, no degradation is triggered because the interaction is “sequence non-specific” and only limited interactions between piRNAs and maternal mRNAs are established that are insufficient to induce cleavage. Because germline mRNAs are longer and more abundant than other mRNAs, partial base-pairing with a larger number of piRNAs is proposed to achieve preferential tethering to the germ plasm together with Aub [76]. Thus, depending on the degree of complementarity, Aub-piRNA complexes can target maternal mRNAs for degradation in the ooplasm/soma or assist in their (intact) localization to the germ plasm. In addition, while Aub recruits the CCR4-NOT deadenylation complex in the soma, it stabilizes mRNAs in the germ plasm through interactions with Wispy poly(A) polymerase [77].

During early embryogenesis, maternal Piwi, Aub and Ago3 are essential for the maintenance of chromatin structure during early mitotic divisions [78]. A role for maternal Piwi has also been implicated in the germline development of the female progeny, suggesting an involvement in germline sex determination [79]. These new functions may be based on the regulation of cellular (non-transposon) genes by the piRNA pathway [79].

#### 2.3.2. Functional Roles of *Drosophila* Piwi Proteins during Germ Cell Differentiation in the Testis

Aub and Ago3 associate with distinct piRNA populations during spermatogenesis. While most piRNAs bound to Aub are derived from the *Su(Ste)* and the *AT-chX* loci and correspond to the antisense orientation, the majority of piRNAs associated with Ago3 correspond to transposons [46]. Furthermore, transposon piRNAs clearly show a ping-pong signature while both *Su(Ste)* and *AT-chX-1* piRNAs do not seem to be produced by a canonical ping-pong mechanism that involves Aub and Ago3 [46]. Aub and Ago3 have also different expression patterns in the male germline: Aub is expressed in all stages, including differentiating primary spermatocytes until meiosis, while Ago3 (and Piwi as well) is restricted to stem cells and spermatogonia, which undergo mitotic divisions [44]. Thus, all three Piwi proteins seem involved in transposon repression in germline stem cells and spermatogonia, while Aub has a role in the regulation of cellular gene expression during spermatocyte differentiation (which may include a “homotypic” mechanism of ping-pong amplification; [44]). 

With respect to the regulation of cellular gene expression by Aub, piRNA clusters were identified that contain many local repeat sequences (as opposed to unique sequences) that produce piRNAs that have the potential to target protein-encoding genes during spermatogenesis [11]. More specifically, using a cutoff of 3 mismatches, it was estimated that 13.8% of testis piRNAs are oriented in antisense to sequences in protein-coding genes. One cluster is located between two paralogous *Hsp70B* genes on chromosome 3 of which piRNAs target the *no distributive disjunction* (*nod*) gene, which is necessary for chromosome segregation during meiosis [80]. Another cluster, *petrel*, is located on the Y chromosome and produces abundant piRNAs highly complementary to the *CG12717* gene (renamed to *pirate*), which encodes a SUMO protease [11]. Direct evidence for targeting of *pirate* by *petrel* was obtained by the identification of a strong ping-pong signature between *pirate* sense piRNAs and *petrel* antisense piRNAs and sequencing of the degradome of the testis. As is the case for Stellate, *pirate* becomes expressed in differentiating spermatocytes following de-repression, indicating its importance in the regulation of spermatogenesis and fertility. 

### 2.4. Functions of Drosophila Piwi Proteins and piRNAs Outside the Gonad

Since piRNAs and Piwi proteins are mainly restricted to the gonads in *Drosophila*, the question arises about the mechanism of transposon control in non-gonadal tissues. In Schneider 2 (S2) cells and fly heads, endo-siRNAs are produced by Dicer-2 from transposons and satellite repeats in the genome [81,82,83]. The precursors of the endo-siRNAs are derived from structured loci and from convergent transcription units and therefore typically have extended dsRNA structures. Knockdown of the genes of the siRNA pathway genes *ago2* and *dcr2* (encoding Dicer-2) resulted in the increased expression of transposons, indicating a role for the endo-siRNA pathway in the control of mobile elements in non-gonadal cells. The endo-siRNA pathway however differs from the exo-siRNA pathway because of the requirement of the dsRNA-binding protein Loquacious instead of R2D2, which functions as a partner of Dicer-2 in the control of viral infections [81,84]. Besides mapping to transposons, a considerable fraction of endo-siRNAs also corresponded to mRNAs, indicating a role in the regulation of gene expression. A considerable fraction of endo-siRNAs mapping to mRNAs is derived from complementary overlapping (convergent) transcripts, particularly in the 3′-UTR regions [81,82].

Although somatic tissues in Drosophila rely on the endo-siRNA pathway for the control of transposable elements, the piRNA pathway nevertheless is expressed in distinct somatic tissues outside the gonads [85]. Nuclear Piwi was shown to interact with heterochromatin protein 1a (HP1a) in the nuclei of cells of the larval salivary glands at both euchromatin and heterochromatin bands in the chromosomes [86]. In the fat body of the adult fly, an active primary piRNA pathway was demonstrated: Piwi was detected in the nuclei of fat body cells; piRNAs that map to transposons and the 3′-UTR of cellular genes were detected by sequencing; and transposable elements became de-repressed in Piwi mutants [87]. Interestingly, re-activation of transposable elements in fat body tissue was associated with the disruption of metabolic homeostasis, leading to a shortening of lifespan [87].

In the brain, Aub and Ago3 (but not Piwi) were expressed in different structures, including divisions of the mushroom body [88]. In both siRNA and piRNA mutants, elevated expression of transposons in the brain could be observed. Mobile transposons were found to insert preferentially at sites near cellular genes that regulate neuronal function in brain tissue, inducing genomic heterogeneity in individual neurons and contributing to neural diversity. Alternative expression of Aub and Ago3 may be important for the regulation of plasticity and integrative properties of mushroom body neurons by a mechanism that involves the control of mobilization of transposons [88]. Besides modulating neuronal function and plasticity, Piwi proteins also play a role in the regulation of cell proliferation in the *Drosophila* brain. Analysis of gene expression in larval brains of *lethal (3) malignant brain tumor* (*l(3)mbt*) mutants revealed the upregulation of genes in the piRNA pathway, including Aub, Ago3 and Piwi [89]. Brain overgrowth is rescued in double mutants of *l(3)mbt* with Piwi and Aub, indicating that the induced expression of Piwi and Aub contributes to brain tumor growth, a property which may be related to their regulation of stem cell proliferation [85,89].

While cell lines from the *Drosophila* ovary such as ovarian somatic sheet cells (OSS; [90]) and ovarian somatic cells (OSC; [62]) harbor an active primary piRNA pathway, this is not the case for cell lines from other tissues [91], including S2 cells that are widely used in RNAi screens [92]. Interestingly, Kc167 cells, which express hemocyte cell markers, also contain an intact canonical primary piRNA pathway [93]. However, Kc167 cells express mainly Piwi and Aub and only very low levels of Ago3 and transcripts from piRNA clusters. As a consequence, few piRNAs are derived from transposons and the majority corresponds to host mRNAs and tRNAs, suggesting that piRNAs are produced from random acquisition of transcripts by the primary piRNA biogenesis machinery [93].

## 3. Insights from Research on Piwi Proteins and piRNAs in Mosquitoes

### 3.1. Diverisification of Piwi Proteins in Aedes Mosquitoes

While in *Drosophila* the piRNA pathway is mainly expressed in the gonads and has major functions in the regulation of germline stem cell maintenance and transposon mobilization in both ovaries and testes [94], in mosquitoes a prominent role for piRNAs and Piwi proteins was found in different somatic tissues and cell lines with respect to the control of viral infections [13,95,96]. Although primary viral piRNAs were also reported for RNA viruses in a *Drosophila* ovarian somatic sheet cell line [97], an extensive and detailed study later established that viral infections are not controlled by the piRNA pathway in *Drosophila* flies [13,98].

Most research has been carried out in the yellow fever mosquito, *Aedes aegypti*, and its derived cell line Aag2 [99]. The genomes of *Ae. aegypti* and *Ae. albopictus* contain eight and seven Piwi-related genes respectively, of which Ago3 clusters with *Drosophila* Ago3 while Piwi1/3 to Piwi7 are considered as an expansion of a hypothetical “Aub/Piwi” ancestral gene (which gave rise to the duplication in Aub and Piwi in Drosophila [16,100,101]. However, while transposon sequences are abundant in the genome of *Ae. aegypti*, only 1/4th–1/5th of piRNAs map to transposable elements [102,103], indicating additional functions in the regulation of expression of cellular genes and antiviral defense [103]. Also in the Asian tiger mosquito, *Ae. albopictus*, piRNA clusters were slightly depleted of transposable elements compared to the rest of the genome [104]. In both *Ae. aegypti* and *albopictus* genomes, piRNA clusters were enriched for the presence of non-retroviral integrated RNA virus elements (NIRVs) or, using a more general term, endogenous viral elements (EVEs) [103,104,105,106].

Diversification of the Piwi subclass proteins in mosquitoes has been associated with functional specialization with respect to their targets. In Aag2 cells, mainly Piwi4, Piwi5, Piwi6 and Ago3 are expressed, which are also the Piwi proteins that are predominant in the somatic tissues of adult mosquitoes [107]. By contrast, Piwi1/3 is germline-specific and Piwi7 is enriched in the early embryo [108]. Immunoprecipitation followed by mass spectrometry identified factors that specifically interact with individual Piwi proteins [109], confirming the specialization of function that is consistent with the diversification of the piRNA pathway. 

### 3.2. The piRNA Pathway as an Antiviral Defense Mechanism in Aedes Mosquitoes

Following infection of mosquitoes and Aag2 cells with RNA viruses such as alphaviruses, flaviviruses and bunyaviruses, both viral siRNAs (vsiRNAs) and viral piRNAs (vpiRNAs) are typically produced [12,13]. Most of the vpiRNAs are derived from the (+) RNA strands of the viruses and show A10 bias, indicating an active ping-pong amplification mechanism. In the case of infection of Aag2 cells with Sindbis virus (*Alphavirus*), knockdown of individual Piwi proteins by RNAi showed that Piwi5 and Ago3 represent the main Piwi proteins that process viral genomes/antigenomes into 25–30 nt vpiRNAs [107]. Immunoprecipitation experiments demonstrated that vpiRNAs (with A10 bias) from the (+) strand preferentially associate with Ago3 while the (−) vpiRNAs (with 1U bias) are mainly bound by Piwi5. Thus, in *Ae. aegypti*, Piwi5 and Ago3 are the core components of the ping-pong amplification mechanism that generates vpiRNAs following Sindbis virus infection [107]. Piwi4, on the other hand, was not found to be required for the ping-pong amplification mechanism [110]. Interestingly, knockdown of Piwi4 can result in an increase of production of primary/initiator piRNAs from piRNA clusters that is controlled by Piwi5, indicating competition between the actions of both Piwi proteins [110]. Specific interaction of Piwi5 (but not Ago3, Piwi4 or Piwi6) with Zucchini also indicates its predominant role in phased piRNA biogenesis [110].

Processing of vpiRNAs by a ping-pong mechanism was found to be localized in cytoplasmic foci known as Ven-bodies, which resemble the Yb bodies and nuage in *Drosophila* somatic and germline cells, respectively. Formation of Ven-bodies is dependent on the Tudor protein Veneno that interacts directly with Ago3 and recruits Piwi5 via the mosquito homolog of Yb [111]. In addition, staining for Attari-PB, a Tudor-domain protein and putative *Drosophila* Krimper ortholog that serves as a scaffold for ping-pong amplification, also detects cytoplasmic foci together with Piwi5 and Ago3 that border with and partially overlap with Ven-bodies [109]. It is suggested that Ven-bodies and Attari-PB granules may constitute sites that represent different steps of piRNA biogenesis [109].

With respect to antiviral defense, however, only knockdown of Piwi4 resulted in increased replication of diverse arboviruses such as the alphaviruses Semliki Forest virus and Chikungunya virus [112,113,114], the flavivirus Zika virus [115] and the bunyavirus Rift Valley Fever virus [116]. Knockdown of Piwi5, Piwi6 or Ago3 had only minor effects despite the preferential association of vpiRNAs with Piwi5, Piwi6 and Ago3 [107,117]. The latter observation argues against a role for the ping-pong amplification loop in antiviral defense [118].

A way out of this conundrum was provided by the observation that Piwi4 is required for the maturation of vpiRNAs by regulating their 3′-end methylation and that Piwi4 immunoprecipitates are enriched with mature vpiRNAs (i.e., that are methylated and resistant to β-elimination) [96]. Furthermore, Piwi4 was identified as a hub protein in the RNAi defense mechanism against RNA virus infections by its essential role in the 3′-end methylation of both vsiRNAs and vpiRNAs and its interaction with Ago2, which is the effector of antiviral immunity by the siRNA pathway [96]. Induction of the expression of Piwi4 in the midgut and carcass was observed following a blood meal, underlining a pivotal role in protection against oral infection by arboviruses [96]. In *Ae. albopictus*, all Piwi genes become induced in the carcass following a blood meal; further increases were observed for most Piwi genes at four days after infection with Dengue virus and for Piwi6 at 14 days after infection with Chikungunya virus [101]. However, other studies established that Piwi4 was mainly bound by two abundant piRNAs from satellite DNA repeats (see below in Section 3.5) and it remains to be investigated to what extent this association plays a role in the antiviral function of Piwi4, for instance through the regulation of expression of cellular genes [118].

While mature vpiRNAs of antisense orientation and U1 enrichment are preferentially associated with Piwi4, evidence was also obtained that such vpiRNAs can be produced from templates of viral cDNA (vDNA) that result from reverse transcription activity of retrotransposons [96,119,120]. Indeed, administration of inhibitors of reverse transcription results in an increase of viral replication. Thus, a model appears in which RNA (sub)genomes of arboviruses and mosquito-specific RNA viruses are incorporated into replication complexes of retrotransposons (mainly *Ty1*, *Ty3* and *Pao Bel* LTR retrotransposons) to form vDNA after recombination and reverse transcription. Transcripts from circular episomal DNAs in the nucleus are then processed into antisense vpiRNAs that participate in the targeting of viral RNAs in the cytoplasm [96]. vDNAs are also produced from defective RNA virus genomes in *Drosophila* where they function as sources for vsiRNAs and not vpiRNAs, presumably because Piwi proteins are not expressed in most somatic tissues in flies [121]. In Aag2 cells, production of vpiRNAs was also observed to be associated with replicating defective genomes of *Ae. albopictus* densovirus, a ssDNA virus, indicating the possible extension of the antiviral function of piRNAs to DNA viruses [122].

The identification of Piwi4 as a major antiviral effector and its association with vpiRNAs that are derived from vDNA further indicate that the abundant vpiRNAs with ping-pong signature, that are associated with Piwi5, Piwi6 and Ago3 and are directly derived from viral RNA, have no large role in antiviral defense and may reflect further degradation following the action of Piwi4 and Ago2 [96]. The exo-siRNA pathway, with Dicer-2 and Ago2 as characteristic components, nevertheless is considered critically important in antiviral defense, which is most clearly demonstrated by the high levels of RNA virus replication in cell lines that are deficient for Dicer-2 [123]. In the absence of Dicer-2 function, abundant vpiRNAs with ping-pong signature are observed that, besides species with normal length of 26–29 nt, also contain reads of 21 nt which were referred to as “vsi-piRNAs”. The 21 nt vsi-piRNAs with ping-pong signature may be considered as genuine products of the piRNA pathway that become unmasked in the absence of functional Dicer-2 and therefore should be distinguished from true 21 nt vsiRNAs [124].

### 3.3. Endogenous Viral Elements and Immunological Memory in Aedes Mosquitoes

A possible role for piRNAs originating from NIRVs or EVEs in antiviral defense has raised considerable interest [103,105,106]. Since formation of retrotransposon-viral hybrid cDNAs is a common phenomenon, their subsequent integration into chromosomal DNA by integrase activity can also be expected, when the (long-term) persistent character of arbovirus and many mosquito-specific virus infections is taken into account. In Aag2 cells, insertions of flaviviruses (*Flaviviridae*) and rhabdoviruses (*Rhabdoviridae*) are enriched with *Ty3/gypsy* elements while EVEs of the *Chuviridae* correlate with *Pao-Bel* retrotransposons [125]. Moreover, EVEs/NIRVs and LTR retrotransposons are often organized in large loci which function as piRNA clusters that are transcribed in antisense orientation [126,127]. A similar organization of mosquito-specific flavivirus- and rhabdovirus-derived EVEs and their enrichment in piRNA clusters was found in the genome of *Ae. albopictus* [104]. Annotation of the newest reference genome of *Ae. aegypti* identified 252 EVEs, which were mostly derived from *Rhabdoviridae*, followed by *Xinmoviridae*, *Chuviridae* and *Flaviviridae* [128,129].

The piRNA clusters that contain EVEs are transcriptionally active and can produce abundant EVE-derived antisense piRNAs that are preferentially loaded in Piwi4. By contrast, piRNAs that target transposable elements are not enriched in Piwi4. Analogous to the control of transposable elements, it can be hypothesized that piRNAs derived from EVEs can play a role in antiviral defense provided sufficient complementarity exists with invading viral genomes. Activation of a ping-pong mechanism was indeed triggered by a piRNA from a genomic cluster interacting with the Phasi Charoen-like virus that persistently infects Aag2 cells [125]. Furthermore, piRISC complexes containing Piwi4 and EVE-derived piRNAs were found to silence reporter constructs and restrict infections by recombinant Sindbis virus that contained complementary targets [96].

If the occurrence of EVEs in piRNA clusters is related to adaptive immunity, it can be expected that their acquisition occurs variably among different mosquito populations that become exposed to different types of viruses. Five novel EVEs corresponding to mosquito-specific flaviviruses or anpheviruses (*Mononegavirales*) were identified among genomes of 80 *Ae. aegypti* mosquitoes sampled from five geographical populations, indicating that integration events are rare [128]. Moreover, only one new EVE was inserted into a piRNA cluster, which, however, was highly active and contained 13 additional viral integrations. Analyses based on the high-quality reference genome of *Ae. aegypti* indicate that insertions of viral sequences can be complex events and that only a few EVEs contribute to adaptive immunity [128,129]. Efficiency of genome integration of viral sequences may also be virus-specific, since no EVEs specific to alphaviruses could be identified so far, which is consistent with the observation that no integration of viral sequences occurred during persistent infection of *Ae. albopictus* with Chikungunya virus (*Alphavirus*) [130].

The importance of EVEs in antiviral immunity through a piRNA mechanism in a natural population nevertheless was demonstrated for an EVE that showed high sequence identity with a strain of the mosquito-specific flavivirus Cell-Fusing Agent virus (CFAV) [131]. In the presence of the EVE, abundant vpiRNAs were observed following CFAV infection that showed a ping-pong signature with 1U (−) piRNAs derived from the EVE and 10A (+) piRNAs derived from CFAV. Removal of the EVE from the genome abolished the production of secondary vpiRNAs and resulted in higher replication of CFAV mainly in the ovaries, which indicates that EVEs may be necessary to protect the germline and maintain fecundity/fertility against vertically transmitted mosquito-specific viruses [131].

Considerable evidence therefore exists that the piRNA pathway, evolved to control transposon silencing in the majority of Metazoa, was adapted in *Ae. aegypti* mosquitoes for the control of virus infections. In addition, the process of integration of viral sequences into genomic piRNA clusters as EVEs provides a mechanism of immunological memory against future infections by related viruses [132]. However, because protection in the offspring would require integration in germline cells, it can be expected that EVEs provide immunity against mosquito-specific viruses (which are vertically transmitted) rather than against arboviruses [12,133].

### 3.4. Virus- and Mosquito-Dependent Variability of the piRNA Pathway as an Antiviral Defense Mechanism

Despite the solid evidence for *Ae. aegypti*, other studies nevertheless have indicated that the contribution of the piRNA pathway in antiviral defense can be both mosquito- and virus-specific [12,134,135]. Expansion of Piwi genes is not observed in all mosquito species: it occurs in the genomes of *Aedes* and *Culex* species (culicine subclade) but not in the anopheline subclade represented by *Anopheles gambiae* [13,100,136].

Production of vpiRNAs in *Culex* mosquitoes was observed to be variable: while no vpiRNAs were observed during alphavirus and flavivirus infections of *Cx. pipiens* [95], a cell line derived from *Cx. tarsalis* showed production of vpiRNAs corresponding to persistent infection by Phasi Charoen-like virus (*Bunyavirales*) but not after acute infection with West Nile virus (*Flaviviridae*) [135]. The proportion of EVEs is also much larger in the genomes of *Ae. aegypti* and *Ae. albopictus* than in *Cx. quinquefasciatus* and *An. gambiae* [137]. In *Ae. albopictus* U4.4 cells, (primary) vpiRNAs were identified after infection with a “dual-host affiliated” but not with a “classical” mosquito-specific flavivirus [138]. Variations in viral genome organization, patterns of replication and differences in subcellular localization of the virus replication complexes likely influences the extent to which the vpiRNA response becomes integrated in the innate antiviral response [133,135].

### 3.5. Other (Non-Antiviral) Functions of piRNAs in Mosquitoes

The mosquito family of Culicidae is divided into the Culicinae and Anopheline subfamilies that diverge strongly in the size of the genome [139]. Specifically, the genomes of culicine mosquitoes, among which especially *Ae. aegypti* and *Ae. albopictus*, are five- to ten-fold greater in size because of the acquisition of many transposons and repetitive elements [136,139]. Transposon-derived piRNAs accumulate preferentially in germline tissues with LTR retrotransposons as the most prominent group among mosquitoes. While transposon-derived piRNAs are more abundant in culicine mosquitoes, it was also observed that relatively low amounts of piRNAs have (−) strand orientation and that piRNA clusters in mosquitoes have lower transposon density than in *Drosophila* [136]. The majority of piRNAs in mosquitoes do not seem to target transposons and their targets still need to be determined in the future.

Knockdown and immunoprecipitation experiments have investigated the association of piRNAs with specific Piwi proteins leading to the classification of transposable elements into several groups in Aag2 cells [107]. Piwi5 and Ago3 are the main Piwi proteins for the production of piRNAs derived from transposable elements and EVEs [107,125,126]. By contrast, Piwi4 is mainly associated with two piRNAs derived from a satellite DNA locus [140] (see further below) although Piwi4 has also an essential role in the antiviral defense by the regulation of the 3′-end methylation of vpiRNAs [96] (see Section 3.2). Using the same approach, piRNAs of coding genes (8% of total piRNAs) could be categorized in six groups [103]. In one group, piRNAs were derived from genes of the H4 histone family. H4-specific piRNAs were predominant of the sense strand that associated with Ago3, showed the signature of ping-pong amplification, and accumulated during the S phase in the cell cycle, with similar dynamics as H4 mRNAs [103].

Only in culicine mosquitoes are piRNA clusters found that are associated with satellite DNA repeats. Because of synteny across mosquito species of both Culicinae and Anophelinae, the evolution of one particular piRNA cluster with satellite DNA repeats (named Mosquito-Conserved piRNA Cluster Locus or MCpiRCL) could be reconstructed. The acquisition of satellite DNA repeats in this cluster was associated with the production of very high levels of two major piRNAs that have ~20–30 alternating repeats over a region of several kb [136]. Remarkably, some of the culicine satellite DNA piRNAs are conserved with piRNAs in the orthologous piRNA cluster in *An. gambiae* that represents the evolutionary ancient condition. The conservation of the piRNA sequences and their expression throughout gonads, somatic tissues and cell lines indicates broad important functions which could be revealed through the identification of the mRNAs, transposons or viruses that are targeted. It is speculated that the expansion of Piwi proteins in culicine mosquitoes is connected to the acquisition of satellite DNA repeats at piRNA clusters to promote the strong periodic piRNA phasing biogenesis pattern that results in high levels of expression [136]. Interestingly, production of single vpiRNAs at high levels could also be observed during infection with Dengue virus [13], indicating that such mechanism could also play a role during vpiRNA production.

Functional analysis of a satellite DNA repeat locus, that is conserved in the Culicinae but not in the Anopheline subfamily, indeed revealed the production of two tandem repeat-associated piRNAs (tapiRs) at high levels that exclusively associate with Piwi4 [140]. Reporter assays revealed relatively relaxed rules of interaction of tapiRs with targets with only limited base pairing needed to induce silencing. Injection of antisense oligonucleotides targeting tapiR1 in embryos revealed a role in the degradation of maternal transcripts at the zygotic genome activation stage which was essential for normal embryonic development. The massive deregulation of transcripts by inhibition of tapiR1 was dependent on Piwi4 and confirmed its capacity to broadly target a multitude of different targets that regulate many cellular processes [140].

Another abundant piRNA, promiscuous piRNA 1 (propiR1), was found to exist as two length isoforms that were differentially associated with Piwi5 and Piwi4 in Aag2 cells [141]. The main target of propiR1 is a long non-coding RNA, lnc027353, which becomes repressed following zygotic propiR1 expression in mosquito embryos. Interestingly, propiR1 exhibits different silencing properties when associated with Piwi4 or Piwi5 that can involve both slicing-dependent and –independent mechanisms [141]. The regulatory circuit containing propiR1 and lnc027353 is conserved between *Ae. aegypti* and *Ae. albopictus*, underlining its biological significance.

Interestingly, while Piwi4 has a cytoplasmic localization in midgut cells, ovaries contained Piwi4 in both cytoplasmic and nuclear fractions. A nuclear localization domain was identified in a disordered domain at the N-terminus of Piwi4 which confers partial localization to the nucleus in Aag2 cells [142]. Distribution in both cytoplasmic and nuclear compartments was also observed for Piwi4, Piwi5 and Piwi6 in Aag2 cells while Ago3 was exclusively cytoplasmic [109]. The presence in the nucleus indicates that mosquito Piwi proteins can form effector complexes involved in transcriptional gene silencing.

## 4. Insights from Research on Piwi Proteins and piRNAs in the Silkworm

### 4.1. A Silkworm Cell Line as a Model for the Molecular Analysis of the piRNA Pathway

Most research on the biogenesis and function of piRNAs was carried out in *Drosophila* regarding their role in the defense against transposable elements in the ovarian germline and the regulation of gene expression during spermatogenesis (see Section 2; [11,143]). With respect to lepidopteran insects, BmN4 cells, a cell line derived from ovarian tissue of the silkworm, *Bombyx mori*, have emerged as a useful cell culture model to study piRNA biogenesis in considerable detail [14]. Based on studies in *Drosophila* and mammals, it was originally thought that the piRNA pathway was mostly restricted to germline cells where the defense against genome disruption by transposons is much more crucial than in somatic tissues [17]. However, recent findings have indicated that the piRNA mechanism is present in somatic tissues of many arthropod species and that the relative restriction of piRNAs to germline cells in *Drosophila* may rather be an exception among insect species [16]. Consistent with this observation is the detection of piRNAs in other lepidopteran cell lines such as Hi5 (derived from the cabbage looper, *Trichoplusia ni*; see further below) and Sf9 (derived from the fall armyworm, *Spodoptera frugiperda*) [144].

The piRNA pathway has been elucidated in detail in silkworm-derived BmN4 cells with respect to the control of transposable elements in the genome [14]. Silkworm cells (and higher Lepidoptera in general; [145]) contain two Piwi class Argonaute proteins, called Siwi (ortholog of Aubergine of *Drosophila*) and BmAgo3 [146]. By contrast, silkworm cells do not contain a homolog of Piwi, which is involved in transcriptional silencing of transposons in somatic tissues of Drosophila [147]. Although interactions with heterochromatin proteins in the nucleus were also reported [148], Siwi and BmAgo3 are mostly located in the cytoplasm where they are involved in post-transcriptional silencing of transposons.

As is the case in *Drosophila*, primary piRNAs typically originate after transcription of particular loci in the genome, known as piRNA clusters that consist of remnants of transposable elements that are mainly transcribed in antisense orientation [149]. The primary piRNAs of antisense orientation in low abundance are the triggers for the control of transposable elements. The piRNA cluster transcripts are processed by endonucleases such as Zucchini [150] and the resulting piRNA precursors are loaded in Siwi with a preference for uracil at the 5′-end [151]. Siwi and BmAgo3 function subsequently as the two effector Piwi class proteins in the ping-pong amplification loop (with 1U-10A signature in piRNAs originating from complementary strands) that results in the abundant production of secondary/responder piRNAs that are essential for posttranscriptional silencing [9,152]. In the scheme of piRNA biogenesis, Siwi is essential for primary piRISC formation, while BmAgo3 is dispensable. On the other hand, BmAgo3 drives the establishment of the ping-pong amplification loop that generates both Siwi- and BmAgo3-dependent secondary piRISCs [153]. In BmN4 cells, multiple trailing piRNAs are also produced downstream of secondary/responder piRNAs via cleavage by Zucchini or additional Siwi- or BmAgo3-mediated catalysis [154]. 

Because of presence of a robust piRNA mechanism that involves the ping-pong cycle (in contrast to *Drosophila* cell lines such as S2 cells), BmN4 cells have functioned as a model for the elucidation of the mechanism of piRNA biogenesis and function [14]. Research of the piRNA pathway in BmN4 cells has clarified several key steps such as the hierarchy of Siwi and BmAgo3 in the ping-pong cycle [152], the trimming of the 3′-ends of piRNAs after their loading in Siwi (which involves the exonuclease Trimmer and the Tudor-domain protein Papi [151,155,156]), primary Siwi-piRISC formation (which occurs at the outer mitochondrial membrane and requires the helicase Spn-E, the Tudor-domain proteins Qin and Papi and the endonuclease Zucchini [152,157]) and the direct transfer of the 5′ processed transposon products from Siwi to BmAgo3 or from BmAgo3 to Siwi where they will mature to secondary piRNAs (which involves Qin and the DEAD box helicase Vasa for the former and Vreteno, another Tudor protein, and DDX43, another Vasa-like protein, for the latter [147,152,153,158]). Moreover, the crystal structure of Siwi of BmN4 cells was determined and used to elucidate particular aspects of the piRNA mechanism such as the slicing mechanism, the length of piRNAs, and the strong selection for 1U piRNAs [159,160].

Piwi class proteins display a well-defined localization pattern in BmN4 cells. While Siwi mainly shows diffuse staining throughout the cytoplasm, BmAgo3 accumulates together with the Tudor protein Vreteno in perinuclear granules called Ago3 bodies that resemble the “nuage” observed in germ cells of Drosophila and are the sites of secondary piRISC formation [153,161]. Accordingly, Siwi is also present in nuage where it strongly colocalizes with Vasa (as Vasa bodies; [147,152]). Because of their dependence on different cofactors, formation of Siwi- and BmAgo3-dependent piRISC is thought to occur at different nuage granules: Vasa bodies constitute the site for BmAgo3-piRISC biogenesis and Ago3 bodies that are enriched for Vreteno constitute the site for (secondary) Siwi-piRISC formation [162]. 

In addition, components of the piRNA machinery such as the exonuclease Trimmer, the Tudor protein Papi and the endonuclease Zucchini are localized together with Siwi or BmAgo3 on the surface of the mitochondria, the site of primary piRISC production [150,155,157,163]. Thus, two distinct organelles, mitochondria and nuage, function as centers for assembly of primary and secondary piRISC complexes, respectively [153]. 

Several other factors in the piRNA pathway, such as the Tudor-domain protein Tdrd12 (ortholog of BoYb in Drosophila) and the RNA helicase Armitage are also localized in the nuage-like granules of BmN4 cells [164,165]. Different cofactors in the piRNA pathway may define separate nuage organelles that have distinct functions, such as the Mael bodies (characterized by the Maelstrom protein), which play a role in the control of evolutionary young “group I” transposons through the regulation of the activity of primary Siwi-piRISC complexes [162]. Interestingly, Siwi was observed to be shuttling between nuage and other membraneless organelles that are called piP-bodies because of their resemblance with mammalian ribonucleoprotein condensates related to mRNA degradation and the miRNA pathway [166]. The efficient shuttling of Siwi from piP-bodies to nuage requires the RNA helicase Vasa. Disruption of transfer can result in trapping of Siwi and other piRNA factors in piP-bodies and the production of non-canonical piRNAs that are derived from mRNAs instead of transposon RNAs [166].

The lepidopteran cell line Hi5 which is derived from ovarian tissue of the cabbage looper, *T. ni*, shares many properties with BmN4 cells regarding the piRNA pathway [167]. Similar to the silkworm and BmN4 cells, *T. ni* and Hi5 cells encode only two Piwi class proteins, TnPiwi and TnAgo3. More than 70 piRNA clusters were identified in Hi5 cells and 75% of piRNAs mapping to transposons are antisense, suggesting a role in transposon control (see also Section 4.2). Comparison between ovarian tissue and Hi5 cells revealed piRNA clusters unique to Hi5 cells that may have been generated by recent transposition events. Hi5 cell nuclei also contain a (female-specific) W chromosome which is regarded as a large piRNA cluster [167] (see also Section 4.3). When genome editing was used to fuse the endogenous Vasa protein with GFP and HA-tag at the N-terminus, perinuclear nuage-like structures were observed in Hi5 cells after staining with specific antibodies [167].

### 4.2. Control of Transposable Elements by the piRNA Pathway in the Silkworm

Small RNAs with length typical of piRNAs (27–30 nt) are enriched in pupal ovarian and testis tissue of *Bombyx* [168]. Sequencing of piRNA-sized small RNAs from the ovary revealed that 46% corresponded to repeat sequences in the genome and transposons. Most hits were associated with non-LTR retrotransposons followed by LTR retrotransposons and class II DNA transposons and a strong bias was observed for the antisense orientation [168]. In addition, a ping-pong signature was observed between sense and antisense transposon-derived piRNAs. Accordingly, mRNAs of the two Piwi proteins of *Bombyx* were abundantly expressed in the gonads [146,169,170] but could also be readily detected in many other larval and pupal tissues [171], conform to an ancient role of the piRNA pathway in somatic tissues [16]. Indeed, a ping-pong signature was observed for transposon-derived piRNAs in both somatic and germline tissues of the gonads [172]. With respect to transposon-derived piRNAs isolated from whole body tissues, a bias towards antisense piRNAs was observed besides the detection of the ping-pong pairs [173]. In *T. ni*, a similar pattern of preferential expression of TnPiwi and TnAgo3 in the gonads was observed, together with the existence of a fully functional piRNA pathway, including ping-pong amplification, in somatic (thorax) tissue [167].

Increased levels of Siwi and BmAgo3 occurred in the testis during spermatogenesis (4th and 5th instar larvae) and in the ovary during oogenesis (pupae and pharate adults). Staining with specific antibodies showed the presence of Siwi and BmAgo3 in perinuclear nuage-like structures in nurse cells of the ovary and in spermatogonia of the testis [170]. In addition, Siwi protein was detected in the somatic cells of the ovary. Gene disruption of *siwi* or *bmAgo3* in female silkworms resulted in a decrease in specific piRNAs in ovarian tissue while an upregulation of transposable elements was also observed in ΔSiwi mutants [170].

High expression of both Piwi genes was observed during early embryogenesis (0–16 h) [146]. Together with the abundant presence of piRNAs in newly laid eggs, piRISCs were proposed to be maternally deposited in silkworm embryos [172]. Many germline piRNAs from the ovary that become maternally deposited in the eggs have an antisense orientation. When zygotic expression of the mRNAs of the LTR retrotransposons *Pao*, *Yamato* and *Benibana* is induced at 12 h after fertilization, production of secondary piRNAs was observed for these transposons, indicating that the maternally deposited piRNAs can function as triggers for ping-pong amplification and control of retrotransposon mobilization [172].

The content of transposable elements in the *Bombyx* genome is relatively large (up to 46%) and comparable with the mosquito *Ae. aegypti* (47%) [174,175,176]. While short interspersed nuclear elements (SINEs) constitute a major part of the genome (13%), relatively few piRNAs map to SINEs, in contrast to the other transposable elements [168,174]. In addition, many transposons have accumulated on the female-specific W chromosome from which many female-specific piRNAs are produced in ovarian tissue [177]. By contrast, only few transposons, e.g., the DNA transposon mariner, produced more piRNAs in the testis of male silkworms that have ZZ sex chromsomes.

In BmN4 cells, a direct role of the piRNA pathway in the defense against transposable elements was demonstrated. Silencing of *bmAgo3* or *siwi* in BmN4 cells increased the expression of the telomeric non-LTR retro-elements *SART1* and *TRAS1* [169]. In another study [178], knockdown of Siwi resulted in an increase in the de-repression of the LTR retrotransposons *Yamato* and *Kimono*. When BmRNase κ, involved in piRNA precursor cleavage and necessary for robust piRNA production, was knocked down in *Bombyx* embryos or BmN4 cells, consistent upregulation of retrotransposons was observed [179]. Direct experimental evidence of the mechanism of silencing of a transgene by the piRNA pathway was also observed in BmN4 cells. Following insertion of a piggyBac transposon containing a GFP expression cassette in the piRNA cluster *torimochi*, production of piRNAs targeting GFP by a ping-pong amplification mechanism were observed that could mediate the silencing of transfected GFP expression plasmids in *trans* [180]. Interestingly, increased cell density resulted in an increase in expression of Piwi proteins and piRNAs concomitant with a decrease in long transcripts (piRNA precursors) of the piRNA cluster *torimochi* and repression of LTR retrotransposons [178]. The increased piRNA biogenesis seemed to be triggered by physical cell-cell contacts and was not dependent on cell proliferation or changes in the cellular medium such as nutrients or diffusible factors.

In addition, small RNAs with antisense orientation corresponding to sequences of transposons, e.g., the non-LTR retrotransposon *Bm1645*, were found to associate with Ago2 protein, indicating that the endo-siRNA pathway can contribute to the control of transposable elements [181]. In Hi5 cells and *T. ni* thorax, ovaries, and testes, 21–52% of endogenous siRNAs also map to transposons, indicating a role in transposon suppression in both somatic tissues and germlines [167].

Approximately 800 piRNA clusters (amounting to 3.9 Mb) are identified in the newest assembly of the *Bombyx* genome [176]. In contrast to *Drosophila* piRNA clusters that are often associated with heterochromatic marks (histone H3 harboring trimethylated lysine 9 (H3K9me3) and HP1), most piRNA loci in *Bombyx*-derived BmN4 cells are located in euchromatin (containing the markers H3K4me2, H3K4me3 and H3K9ac) and are transcribed by RNA polymerase II as 5′-capped and poly-adenylated transcripts [149]. Telomeric piRNA clusters such as SART1, which are located in constitutive heterochromatin, on the other hand, have both euchromatic and heterochromatic features [149]. 

### 4.3. Sex Determination by the piRNA Pathway in the Silkworm

In silkworms, females constitute the heterogametic sex (ZW) while males have two morphologically similar (ZZ) sex chromosomes. The W chromosome in females consists almost entirely of transposable and repeat elements, including the sex-determining region [177]. A piRNA precursor transcript, called *Feminizer* (*Fem*), corresponding to more than 150 (non-transposable) repeats in the sex-determining region was identified that produces a single piRNA at high levels in female embryos [15,182]. The target of *Fem* piRNA is the gene *Masculinizer* (*Masc*) located on the Z chromosome that encodes a CCCH-tandem Zinc finger protein. Cleavage of *Masc* mRNA leads to the activation of a canonical ping-pong mechanism in which *Fem* piRNAs are bound to Siwi and *Masc* piRNAs preferentially associate with BmAgo3 [15]. RNA-seq and western blot established that *Masc*-piRNA-BmAgo3 complexes are maternally deposited in embryos which then cleave zygotically expressed *Fem* precursor transcripts to unique *Fem* piRNAs in female embryos [183]. Activation of the ping-pong cycle prevents Masc protein expression and the female splicing pattern of *doublesex* (*dsx*) is maintained in female embryos. In the absence of *Fem* piRNAs, the presence of Masc protein will promote male-specific splicing of *dsx* in males [15,184]. The gene *dsx* functions at the base of the sex determination cascade that triggers male or female differentiation in somatic tissues of insects [185].

Gene disruption by the CRISPR/Cas9 system established that deficiency of Siwi but not of BmAgo3 caused failure of oocyte development and partial female to male sex reversal of secondary sexual characteristics [170]. On the other hand, no phenotypic changes were observed in testes and male somatic tissues in both ΔSiwi and ΔBmAgo3 silkworms. The effects of Siwi knockout on sex reversal in females were correlated with the detection of male-specific splicing of *dsx* and an increase in abundance of *Masc* mRNA [170]. Interestingly, BmAsh2, a component of the histone methyltransferase complex that mediates H3K4me3 modifications, interacts with Siwi and co-localizes in nuage-like structures [170]. BmAsh2-knockout silkworms phenocopied *siwi* mutants regarding loss of transposon control and partial sex reversal in females. While H3K4me3 levels were decreased in ΔBmAsh2 silkworms, no effect was observed on Siwi expression, indicating that BmAsh2 did not act upstream of Siwi in sex determination or transposon silencing [170]. Another factor that interacts with Siwi and regulates sex determination and transposon control through the piRNA pathway is the CHHC-type zinc finger protein Gametocyte-specific factor1 (Gtsf1) [186]. Control of *Fem* piRNA biogenesis and Masc repression by Gtsf1 was not only observed in the gonads, but also in fat body and olfactory tissue, and was necessary for proper expression of sex-specific genes in female silkworms. Knockout of Gtsf1 resulted in de-repression of transposons and disrupted both testis and ovarian development [186]. Recent research revealed that Gtsf1 is an auxiliary factor that potentiates the piRNA-guided cleavage activity of Siwi but not BmAgo3 in the silkworm [187]. On the other hand, disruption by the CRISPR/Cas9 system of other homologous genes that were identified in the piRNA pathway in *Drosophila*, e.g., *zucchini* and *papi*, did not result in obvious phenotypes in Bombyx, illustrating the differences between the two insect species [188].

The Masc-dependent sex determination system is unique and conserved in Lepidoptera [188,189]. It is speculated that the *Fem* gene may have originated from a translocation of a *Masc* fragment to the W chromosome [190]. However, the hybridization of the *Fem* piRNA (from *Bombyx*) to *Masc* mRNA is not conserved in *Trilocha varians* which belongs to the Bombycidae family [191] and sequences of the *Masc* gene are not very conserved [167]. Whether the piRNA pathway is involved in sex determination in other Lepidoptera remains to be investigated.

### 4.4. The piRNA Pathway as an Antiviral Defense Mechanism in Lepidoptera

Research on the involvement of the piRNA pathway in antiviral defense in Lepidoptera has mainly been carried out in continuous cell lines derived from *B. mori* (BmN4, Bm5), *T. ni* (Hi5) and *S. frugiperda* (Sf9, Sf21), which were often persistently infected with RNA viruses, such as Flock house virus (*Nodaviridae*), Macula-like latent virus (MLV; related to *Tymoviridae*) and Sf-Rhabdovirus (Sf-RV; *Rhabdoviridae*) [191,192,193,194]. Small RNA-seq typically reveals the presence of predominantly 20 nt viral siRNAs, indicating that the siRNA pathway restricts viral infections [168,195,196,197]. In some cases, small RNAs of 27–32 nt (mostly of sense orientation) were also observed in Hi5 cells but these are not considered piRNAs because of the absence of U1 bias or ping-pong signals [167,195,197].

In BmN4 cells persistently infected with MLV, however, small viral RNAs of 28 nt and 27 nt were found to be bound with Siwi and BmAgo3, respectively [198]. While most viral small RNAs of these sizes were of sense orientation and corresponded to the subgenomic region, they also exhibited some typical features of piRNAs: 1U enrichment for Siwi-bound 28 nt sense RNAs and ping-pong signature for both BmAgo3- and Siwi-bound antisense 27–28 nt RNAs [197,198]. On the other hand, production of such vpiRNAs was not observed in MLV-infected Bombyx VF and Bm5 cells, as well as in other stocks of BmN4 cells that were persistently infected with MLV and Sf-RV. Also, no vpiRNAs were observed in BmN4 cells when an infection with the RNA virus Cricket paralysis virus (CrPV; *Dicistriviridae*) was induced [196,197,198]. Moreover, no vpiRNAs were observed in Hi5 cells persistently infected with MLV- and FHV; as well as after infection of Bm5, BmN4 and Hi5 cells with CrPV [196,197].

Knockdown of siRNA factors such as Dcr2 and Ago2 results in higher levels of MLV replication in Hi5, VF and BmN4 cells, as expected, while their over-expression also partially protects Hi5 cells against pathogenic CrPV infection [198,199]. In addition, higher MLV replication is observed in BmN4 cells but not in VF cells after knockdown of Siwi and BmAgo3, consistent with the production of vpiRNAs in BmN4 cells but not in VF cells [198]. On the other hand, knockdown of Piwi class proteins in BmN4 cells or Hi5 cells was also shown to result in an increase of RNA virus infection in the absence of production of vpiRNAs [197]. Over-expression of (*Bombyx*) Siwi and BmAgo3 in Hi5 cells also decreased CrPV replication and increased cell viability [197] although production of vpiRNAs was never observed during CrPV infection of lepidopteran insects [197,198]. Overall, these observations indicate that Piwi class proteins have an antiviral role in some lepidopteran cell lines, possibly independent of the production of (genuine) vpiRNAs.

Whether Piwi proteins can play a role in the antiviral defense against RNA viruses in the absence of vpiRNAs in lepidopteran insects *in vivo* remains to be further clarified [199]. In this regard, a weak induction of Siwi expression was detected during pathogenic infection of midgut tissue by the Lepidoptera-specific dsRNA virus cytoplasmic polyhedrosis virus (*Cypovirus*, *Reoviridae*) in silkworm larvae, while no production of genuine vpiRNAs characterized by 1U bias and ping-pong signature was observed [200,201].

In addition, during infection of lepidopteran insects with nucleopolyhedroviruses (*Baculoviridae*), vsiRNAs are produced [202] but no vpiRNAs [203]. On the other hand, the population of host piRNAs was found to be altered in fat body and midgut tissue at 24 h after oral infection with *B. mori* nucleopolyhedrovirus (BmNPV) in silkworm larvae [203]. *In silico* target gene prediction for differentially expressed piRNAs revealed the possibility of altered gene regulation in fat body tissue that is related to changes in piRNA expression. Interestingly, an antisense piRNA that targets Siwi becomes downregulated during BmNPV infection, reinforcing the idea of an active involvement of the piRNA pathway during BmNPV infection [203]. In concordance with this, knockdown and over-expression studies indicated that Siwi and BmAgo3 can promote BmNPV replication in silkworm cells, which is, however, consistent with a proviral instead of an antiviral role [204]. During baculovirus infection, a reorganization of the cellular nuclear chromatin takes place concomitant with the formation of the virogenic stroma, the site of virus genome DNA replication [205,206]. Differential expression of transposons during baculovirus infection indeed has been reported [207]. It can be speculated that Piwi proteins are required to protect virus genome replication from a possible increase in transposon expression triggered by the changes in host chromatin [205] and it would therefore be interesting to follow the association of piRNAs with Siwi and BmAgo3 during the baculovirus infection cycle.

Although the content of repetitive and transposable elements in *Ae. aegypti* and *B. mori* is similar (46–47%), the latter genome contains considerably less EVEs (273 *versus* 54; [105]). Nevertheless, EVEs in the Bombyx genome are enriched in piRNA clusters and give rise to piRNAs with a ping-pong signature. While *Bombyx* contains only two Piwi genes, *S. frugiperda* encodes five Piwi members of which two underwent a recent duplication [204]. The number of EVEs in *S. frugiperda* is comparable with *Ae. aegypti* and Rhabdovirus-like EVEs in Sf9 cells are actively transcribed [208]. The possible role of EVEs, which are located in piRNA clusters, with respect to antiviral immunity in Lepidoptera therefore merits further investigation.

### 4.5. piRNAs and the Stress Response in Silkworm Cells

Stress conditions and hormone signaling can trigger the cleavage of tRNAs into “tRNA halves” that play a role in stress granule formation, translation, and cell proliferation [209]. In BmN4 cells, abundant piRNAs were identified from the 5′-halves of specific tRNA species (tRNA-derived piRNAs or td-piRNAs; [210]). More specifically, td-piRNAs derived from tRNA^AspGUC^ and tRNA^HisGUG^ comprised 87% and 97% of the total td-piRNAs bound by Siwi or BmAgo3, respectively. Because of their derivation from single tRNA species, the td-piRNAs belong to the most abundant piRNAs expressed in BmN4 cells [210]. The td-piRNAs do not derive from the tRNAs themselves but from the processed 5′-halves of the tRNAs that are cleaved in the anticodon loop to generate 3′-terminal 2′,3′-cyclic phosphates (3′-cP). Recently, *Bombyx* RNase Kappa was identified as the endonuclease that generates 5′-tRNA halves with 3′-cP termini that can function as precursors for td-piRNAs [179]. Interestingly, BmRNase κ localizes to the mitochondria and was shown to be required for robust piRNA biogenesis that is essential for transposon control. The endonuclease BmRNase κ evidently is involved in the cleavage of primary piRNA precursor molecules (such as piRNA cluster transcripts but also specific tRNAs) into precursor piRNAs that are further processed by primary piRNA pathway components such as Papi, Spn-E, Vasa, and Trimmer [179]. The function of td-piRNAs remains unknown.

## 5. Insights from Research on Piwi Proteins and piRNAs in Other Insects

### 5.1. piRNAs, piRNA Clusters and Piwi Genes in Cockroaches, Termites and Locusts

The genome of the German cockroach, *Blatella germanica*, has a high level of repetitive elements (55%) [211] and produces abundant amounts of small RNAs of the piRNA-size class (26–31 nt) [212]. Most piRNA-like small RNAs mapped to transposons and introns and 40% of total piRNA reads were located at >200 piRNA clusters. Using stringent criteria, secondary piRNAs with ping-pong signature were identified that showed specific patterns during development. Interestingly, expression of groups of piRNAs and piRNA clusters was correlated with particular stages of embryonic and post-embryonic development, e.g., the maternal to zygotic transition early in embryogenesis [212].

Termites are social insects in which large differences exist in age between the reproductive and worker castes. Transcriptome analysis revealed increased expression of transposable elements in aging major workers of the fungus-growing termite *Macrotermes bellicosus* (in contrast to aging minor workers, kings, or queens; [213]). The upregulation of DNA transposons and retrotransposons with age in major workers was correlated with a decrease in expression in genes that are involved in piRNA biogenesis such as Zucchini and Aub1 (the latter is one of the Piwi genes together with Aub2 and Ago3). The dysregulation of the piRNA-related genes and transposons is considered less harmful in this social caste because of their lack of participation in the expansion of the colony population [213].

The genome of the migratory locust, *Locusta migratoria*, is very rich in repetitive elements (approximately 60%) in which DNA transposons (24%) and LINE elements (17%) are abundant [214]. The identification of piRNAs of 28–29 nt with ping-pong signature revealed the presence of an active piRNA pathway that produces secondary (responder) piRNAs [215]. Small RNAs that map to transposons (about 20%) could be divided into two groups of sizes 22–23 nt and 26–29 nt, corresponding to endo-siRNAs and piRNAs. Interestingly, the endo-siRNAs and piRNA-like small RNAs are enriched in locusts of the solitary phase compared to the gregarious phase, perhaps reflecting a role in the regulation of epigenetic changes that underlie phase transition in the locust [215,216].

Piwi genes have expanded slightly in *L. migratoria* [16] to one gene encoding Ago3 and two genes (Piwi1 and Piwi2) that correspond to Aub/Piwi of *Drosophila* [217]. Piwi genes in the locust are most highly expressed in the gonads and expression of Piwi2 is pronounced in the testis [217]. However, significant expression of Ago3 and Piwi1 is observed in all somatic tissues, notably the brain. In the brain, significant levels of small RNAs, derived from transposons, are also detected, that have the hallmarks of piRNAs (25–27 nt size, 1U bias, ping-pong signature) [217]. 

Knockdown of Piwi resulted in elevated expression of a substantial number of transposons [218]. In addition, silencing of Piwi had a substantial impact on the expression of protein-encoding genes, which was related to the prevalence of sequences of transposons into the transcripts that were upregulated [218]. In the locust, high levels of “exonization” of transposable elements are observed, i.e., their transposition into introns followed by their recruitment as exons in transcripts. The incorporation of transposon sequences is detected mostly in UTRs and non-coding RNAs and creates transcripts that become prone to regulation by the piRNA pathway [218]. 

The migratory locust has a very large genome size in which also the sizes of the introns have expanded considerably. The presence of large genes in the genome may have stimulated a new nuclear function of piRNAs, i.e., in the processing of large introns (>200 kb) by recursive splicing [217]. A prominent phenotype of knockdown of Piwi1 (but not Ago3) is the loss of weight caused by a decrease in food intake that was correlated with a decrease in expression of the *npf1*, which encodes neuropeptide F, a peptide hormone that is expressed in neuronal tissue and is involved in the regulation of food uptake [219]. Small RNA sequencing identified two piRNAs (piRs-3-I2 and piRs-3-I3; of sense orientation) that located in the second intron of *npf1*, were associated with Piwi1 and could rescue the phenotype of Piwi1 knockdown after exogenous application [217]. Functional assays indicated that piRs-3-I3 stimulated splicing efficiency by interfering with the formation of a hairpin structure at the branchpoint site in the intron that presumably affected assembly of the splicing machinery [217]. A nuclear function of Piwi proteins has been well documented in *Drosophila* in which piRNA complexes deposit H3K9me3 modifications to establish repressive chromatin [9]. The epigenetic modifications by Piwi in Drosophila do not only result in changes in transcription but have also been documented to regulate splicing [220]. In the case of *Locusta*, however, splicing regulation does not seem to be accomplished by chromatin modifications but by specific interactions with nucleic acid structures in unspliced precursor mRNAs.

### 5.2. The Piwi Pathway in Hemiptera: Defense against Parasitic Elements in Whiteflies and Regulation of Reproductive Plasticity in Aphids

About 40–45% of the whitefly (*Bemisia tabaci*) genome consists of repeat and transposable elements that include LINEs, SINEs, LTR retrotransposons, DNA transposons and miniature inverted repeat transposable elements (MITEs) [221,222]. Four Piwi genes (Ago3 and three Piwi-like genes) are present in the *Bemisia* genome and small RNA-seq indicated that reads of 28–31 nt, consistent with the length of piRNAs, were the most abundant [223,224]. Approximately 19% of piRNA-like small RNAs mapped to repetitive elements while the majority corresponded to non-coding sequences (intergenic regions, introns, UTRs). A large number (57–96) of piRNA clusters were identified in the whitefly genome that, among transposons, represented predominantly MITEs (21–23%) followed by LTR elements (9–10%), non-LTR elements (6–8%) and DNA transposons (6%) [223]. Interestingly, piRNA clusters were identified that become induced or suppressed following infection with the begomovirus tomato yellow leaf curl virus (TYLCV) (*Geminiviridae*). The piRNA clusters corresponded to protein-coding genes and transposable elements [223].

Large amounts of piRNAs are found in the somatic tissues of *Bemisia* that show 10 nt overlap (ping-pong signature) and trailing 1U reads (phasing pattern) consistent with the ubiquitous expression of Piwi3 and Ago3 (Piwi2 likely is preferentially expressed in the gonads while Piwi1 has low expression; [224]). The piRNA clusters are also active in somatic tissues and their targeting properties suggest not only a role in genome surveillance but also in gene regulatory networks [224]. Although many clusters generate endo-siRNAs together with piRNAs, production of piRNAs nevertheless was predominant in somatic tissues, including midgut tissue, and it was therefore investigated whether the piRNA pathway could be exploited as a strategy for pest control. Feeding experiments indeed showed that ingested RNAs can enter the piRNA pathway and trigger silencing with similar efficiency as dsRNAs in the siRNA pathway [224]. To increase the likelihood of entrance into the piRNA pathway, target sequences were fused at both ends with sequences from piRNA clusters that showed high expression and delivered as both ssRNA and dsRNA. Following feeding, processing by both siRNA (Dicer processing) and piRNA (ping-pong signal, phasing) pathways was observed that correlated with gene silencing. Thus, exploitation of the high activity of the piRNA pathway in the somatic tissues of the whitefly may stimulate the development of methods for improved control by RNAi-based strategies [224,225].

In the Asian citrus psyllid, *Diaphorina citri*, 27–32 nt vpiRNAs, characterized by 1U/10A bias and ping-pong signal, were observed during persistent infection with the DNA virus D. citri densovirus (DcDNV; *Parvoviridae*) although their abundance was much lower than vsiRNAs [226]. By contrast, no vpiRNAs were detected after infection with RNA viruses specific to *D. citri* (representing reoviruses, bunyaviruses, picorna-like viruses and flavi-like viruses). Interestingly, some populations of *D. citri* also carry a DcDNV-derived EVE in a piRNA cluster of the genome that shares 86% identity with DcDNV and produces EVE-derived piRNAs in all tissues but with highest levels in the midgut [226]. When EVE-harboring psyllids were infected with CrPV engineered to carry an antisense sequence to the EVE, however, no evidence was obtained for the production of vpiRNAs with ping-pong signal that target the recombinant region [226]. This result was observed after both intrathoracic injection and oral acquisition indicating that primary piRNAs derived from the EVE-containing piRNA cluster are not capable to target complementary sequences in recombinant CrPV RNA during infection. The *D. citri* genome contains only two Piwi genes (Ago3 and Aub/Piwi; [227]) and therefore lacks the expansion of Piwi genes that is observed in mosquitoes and that is thought to underlie the expansion of the piRNA pathway to antiviral defense.

Aphids stand out for their capacity to adapt their phenotype to environmental conditions, called phenotypic plasticity [228]. In response to the photoperiod, parthenogenetic viviparous females develop embryos/larvae that either continue the asexual cycle (as virginoparae; in spring and summer) or give rise to offspring of males and oviparous females (as sexuparae; in autumn) [229,230]. Aphid genomes are characterized by a high level of gene duplication and expansion that is thought to underlie the developmental plasticity and polymorphisms [231,232]. A large expansion of Piwi-class genes is observed in the pea aphid, *Acyrthosiphon pisum*, amounting to eight Piwi-like genes and two Ago3 genes [233]. Asexual and sexual morphs differ most significantly in the process of oogenesis: in parthenogenetic females, meiosis is modified to a single reduction division without loss of heterozygosity and oocyte determination/differentiation is succeeded (within the ovary) by embryogenesis in the absence of yolk uptake (vitellogenesis) and chorion formation [234,235]. In sexual females, on the other hand, oogenesis closely resembles the classical mode of meroistic telotrophic development in other hemipterans. Males are determined by the removal of one X chromosome during meiosis [234].

In the pea aphid, Piwi-like proteins are divided in two groups (Piwi1/4/7/8 and Piwi2/3/5/6) that can be further subdivided to closely related pairs (Piwi1/7, Piw2/5 and Piwi3/6). Expression analysis indicates the specialization of Piwi proteins: Piwi2 and 6 are preferentially expressed in the germline in contrast to somatic expression by closely related Piwi5 and 3, respectively; Piwi6 and Ago3b are strongly enriched in sexuparae; Piwi5 is highest expressed in males [233]. Piwi proteins display unique patterns of expression during oogenesis (in the trophic core, prospective oocytes, and oocytes), early embryogenesis (as maternal mRNAs) and late embryogenesis (e.g., associated with germ cell development) [233], indicating roles in the diversification of the reproductive modes in the pea aphid. Analysis of the transcriptomes of asexual and sexual embryos indicated differential gene expression associated with cell cycle, epigenetic regulation, and RNA maturation [236]. Similarly, extensive differential splicing can be found among different reproductive morphs, for instance in microtubule-associated proteins that transport maternal mRNAs into the oocyte [237]. Unfortunately, no comprehensive analysis of piRNAs of the pea aphid was performed and their binding to specific Piwi proteins has not been reported. For instance, during accelerated parthenogenetic development, maternal deposition of piRNA complexes implicated in transposon defense seems wasteful since no invasion of paternal transposons during development is expected. Alternation between sexual and asexual phases is also associated with expansion of Piwi genes in ciliate protozoa and the crustacean *Daphnia* [238,239], strengthening the hypothesis for a specific function for the piRNA pathway.

In another hemipteran insect, the blood-feeding bug and vector for Chagas disease, *Rhodnius prolixus*, a more modest number of Piwi genes are found (three Piwi-like genes and one Ago3 ortholog) [240]. In previtellogenic ovaries, *in situ* hybridization showed that Piwi2 was expressed in both germline and somatic epithelial cells and that Piwi3 and Ago3 were restricted to the germline while no Piwi1 expression was detected. Interestingly, Piwi2 transcripts accumulated at the anterior pole of the oocyte and Piwi2 protein is predicted to feature a PolyQ tract at its N-terminus which is conserved among other Triatomine species but not observed in other insects. Transcripts of all Piwi genes, with the exception of Piwi1, were also found in mature eggs, indicating a role as maternal mRNAs [240]. Injection of dsRNA targeting Piwi genes in females disrupted oogenesis and embryogenesis of which the most severe phenotypes were found following knockdown of Piwi2. Ovaries from Piwi2-knockdown females displayed signs of DNA damage in the zone of germ cell proliferation (likely caused by mobilization of transposons), accumulation of nuclear debris in the trophic core and oocyte collapse during vitellogenesis [240]. Further exploration of the role of the Piwi genes in transposon control needs detection of piRNAs by sequencing and their mapping to transposable elements (which form 6% of the Rhodnius genome of which the majority consists of *mariner* DNA transposons; [241]). On the other hand, during transovarial transmission of persistently infecting RNA viruses, only vsiRNAs and no vpiRNAs were detected, indicating that the piRNA pathway is not involved in antiviral defense [242].

### 5.3. piRNAs, piRNA Clusters and Piwi Genes in Beetles (Coleoptera)

The red flour beetle, *Tribolium castaneum*, encodes only the basic set of two Piwi genes, Piwi/Aub and Ago3 [243]. Small RNAs with the size of piRNAs (26–32 nt) are abundant in oocytes and throughout embryonic development [244]. As in other insect species, a large number (187) of discrete loci were identified that functioned as sources of abundant piRNAs (piRNA clusters) [245]. Transposons of various classes (LTR and non-LTR retrotransposons, DNA transposons) are enriched in piRNA clusters and 70% of transposon-mapped reads were in antisense orientation. Evidence indicates that piRNA clusters are uni-stranded and produce abundant primary piRNAs (with 1U bias) that are deposited in embryos as maternal piRNAs [245]. When zygotic transcription is activated, activation of the ping-pong amplification mechanism is observed concomitant with an increase in transposon expression that therefore seems to function as a defense mechanism against transposon mobilization. On the other hand, piRNAs generally did not map to regions of canonical genes such as UTRs [245]. In contrast to Drosophila, high levels of piRNAs are maintained at all stages of embryogenesis, consistent with their role in somatic tissues. Similar to piRNAs, mRNAs of Piwi/Aub and Ago3 are not only maternally deposited but also expressed zygotically throughout the early blastoderm. Expression of the two Piwi genes also seems to diverge at later stages of embryogenesis with Ago3 more highly expressed in the embryonic body and Piwi/Aub in the serosa [245].

Although the genome of the rice weevil, *Sitophilus oryzae*, contains a considerably larger amount of repeat and transposable elements than Tribolium (approximately 70% versus 50%; [246,247]), no expansion of Piwi genes was detected. The genes encoding piRNA factors, including Piwi/Aub and Ago3, are most highly expressed at the apexes of the ovaries (where oocytes and intracellular symbiotic bacteria are located) [247]. Much lower levels were detected in testis and midgut tissue.

### 5.4. piRNAs, piRNA Clusters and Piwi Genes in Bees (Hymenoptera)

As is the case for the silkworm and *Tribolium*, the honeybee, *Apis mellifera*, encodes two Piwi proteins, Piwi/Aub and Ago3 [248]. Interestingly, the transposable content in the genome of the honeybee is very low (3%), making this species an interesting case with respect to the importance of the piRNA pathway for transposon control [249]. Consistent with a critical role in the protection of genome integrity in the germline, the expression of Piwi genes and piRNAs is highest in males or drones (dominant effects are expected in haploid individuals), intermediate in reproductive queens and lowest in sterile workers [248,250].

Tissue-specific expression was observed with high abundance of defined piRNAs in ovaries, testes, sperm, and brain. The high abundance of piRNAs mapping to transposons in both ovaries and eggs suggests maternal deposition of piRNAs as protection against transposon activation during embryogenesis. Among the different classes of small RNAs (which also include miRNAs and tRNA fragments), piRNAs showed the greatest divergence between eggs from mated queens and eggs from virgin queens [251].

The piRNAs that map to transposons (mainly the DNA transposons Mariner and PiggyBac and the non-LTR retrotransposon R2) show the hallmarks of both the primary pathway (antisense orientation, 1U bias) and the ping-pong mechanism. In reproductive tissue, however, the majority of piRNAs seem to arise by the primary pathway [251]. A majority of the transposon-associated piRNAs can be mapped to piRNA clusters of which many are differentially active in drones, queens, and workers [250]. The piRNAs derived from piRNA clusters also show a phased pattern indicative of the production of trailer piRNAs, especially in the semen [251]. Furthermore, the piRNA clusters have a strong tissue-specific expression. In ovary tissue, more than 50% of piRNAs derive from piRNA cluster 83 that mainly contains retrotransposon fragments [251]. In contrast to ovarian tissue, piRNAs in the semen principally mapped to cluster 8 and contained remnants of DNA transposons together with the LINE retrotransposon R2.

However, 70% of piRNAs were mapped to introns and intergenic regions (although possibly corresponding to mutated or unknown transposable elements) while also a large number associated with protein-coding genes in a tissue-specific pattern. In addition, semen piRNAs that target protein-encoding genes differ from those in other tissues since they align in the sense orientation to exons instead of introns [251]. Interestingly, piRNA cluster 20 overlaps with the genes *complementary sex determiner* (*csd*) and *feminizer* (*fem*), suggesting a role for piRNAs in sex determination.

While piRNAs were clearly detectable in the testis of the honeybee, male germline piRNAs are absent in the bumblebee, *Bombus terrestris* [16]. Since also only low levels of Piwi and Vasa are detected in the bumblebee testis, the piRNA pathway may not be functional in the male germline of the bumblebee. Male bumblebees (as honeybee drones) are haploid, and spermatogenesis therefore proceeds by mitosis rather than meiosis, which could have favored the emergence of an alternative mechanism of transposon control, at least in the bumblebee.

During viral infections, no vpiRNAs were identified in the honeybee [252] or the bumblebee [195], indicating that the piRNA pathway does not contribute to antiviral defense in hymenopterans. Interestingly, EVEs can be found in hymenopteran genomes, for instance EVEs derived from virga/nege-like viruses in bumblebees and ants [253]. With respect to the honeybee, it was reported that 30% of the populations carry an EVE corresponding to Israeli acute paralysis virus (IAPV; *Dicistroviridae*) of which the presence was associated with resistance to virus infection [254]. While the IAPV-derived EVE is transcribed, its colocalization with a piRNA cluster or capacity for production of piRNAs has not been documented.

## 6. Discussion

An overview of the piRNA pathway and its functions in insects is presented in Figure 1 and Table 1. Below follows a general analysis of the peculiar properties of the piRNA pathway and the mechanisms by which its function can be extended from transposon control to gene regulation. Finally, the role of piRNAs and Piwi proteins in stem cell function was reconsidered.

### 6.1. A Universal piRNA Pathway

The silencing of transposons in the germline is considered the ancestral function of the piRNA pathway [28]. In arthropods, Piwi proteins and piRNAs also ensure genome integrity against transposon mobilization in somatic tissues [16]. Although significant diversity was initially perceived, a single piRNA biogenesis pathway is now proposed that is conserved among all animals [8]. Biogenesis of piRNAs is initiated by the transcription of long single-stranded RNAs, often of antisense orientation to transposon sequences, from genomic loci called piRNA clusters [7]. In the germline of *Drosophila,* dual-strand piRNA clusters are located in the heterochromatin and are transcribed by the recently evolved (drosophilid-specific) Rhino-Deadlock-Cutoff complex [255]. In the soma of *Drosophila* and tissues of other insects, however, uni-strand clusters dominate that are presumed to be transcribed by RNA polymerase II (to produce mRNA-like transcripts that are capped, spliced and poly-adenylated; [136,149,245,255]).

In the primary (initiator) pathway, the long piRNA precursor transcripts are transported to the cytoplasm and fragmented by endonucleolytic activity, such as the Zucchini endonuclease that is localized at the mitochondria. However, piRISC complexes that are loaded with complementary piRNAs (which are secondary piRNAs, see next paragraph), for instance derived from transposons, can also cleave the primary piRNA transcripts [28]. The endonucleolytic activity subsequently can extend at regular intervals along the primary transcript in the 3′-direction to produce head-to-tail phased (trailing) precursor piRNAs [8].

In the presence of RNAs of opposite orientation to the piRNA cluster transcripts, typically after transposon expression, the ping-pong amplification loop is activated. Cleavage of transposon transcripts by primary piRISC complexes generates many secondary (responder) piRNAs of opposite orientation that are loaded into Ago3-piRISC complexes to amplify antisense piRNA production [9,28]. Detection of the ping-pong signal generally is considered indicative of silencing following target expression [172]. While the production of secondary piRNAs occurs in nuage-like structures, the Armitage helicase has been implicated in the shuttling of piRNAs between nuage and mitochondria to couple ping-pong amplification with phased piRNA production [30].

In the biogenesis model, maturation of the precursor piRNAs, which includes trimming and 2′-OH methylation of the free 3′-ends, occurs in complex with Piwi proteins through binding of the 5′-phosphate. An important corollary therefore is that the cellular concentration of piRNAs is similar to that of the Piwi proteins, which was verified during spermatocyte differentiation in the mouse testis [8]. Thus, it can be expected that the expression levels of Piwi proteins, especially Piwi/Aub that usually is loaded with piRNAs that are antisense to transposons, reflect the importance of the silencing mechanism in the cells.

### 6.2. Rules for piRNA-Target Interactions

The slicing activity of Piwi proteins is much slower than that of other Argonaute proteins [187] and generally requires an auxiliary factor, the small CHHC zinc-finger protein Gtsf1, to enhance its efficiency [186,256]. Gtsf1 paralogues are distinguished by their interaction with Piwi paralogues; in the silkworm, Gtsf1 increases the rate of cleavage of Siwi but not of BmAgo3 [187]. Efficient target cleavage by Piwi proteins also requires extensive base-pairing with their targets (21 bp) that considerably surpasses that of Argonaute proteins (minimum of 11 bp for *Drosophila* Ago2; [257]). Following Gtsf1-stimulated target cleavage, the release of the cleaved products is slow and considered as the rate-limiting step [187]. The tendency of piRISC to remain bound to the cleavage products can be considered beneficial for the transfer to Piwi partners in the ping-pong amplification loop [152].

Because piRNAs function as guides to identify transcripts for degradation, the targeting mechanism of piRNAs has been compared to that of the miRNAs that operate as prominent post-transcriptional regulators of cellular gene expression [2,258]. However, there are fundamental differences in the rules of target interaction between miRNAs and piRNAs [259]. In miRNAs, the seed-region that defines the initial interaction is pre-organized which allows efficient engagement with the target [260]. While a seed region is also present in piRNAs, base-pairing is much weaker which results in lower target affinity [259]. Stable target association therefore requires additional base-pairing, which increases the fidelity of target interaction. The requirement for extended target complementarity to achieve efficient target cleavage also results in higher stringency. Beyond the seed region, however, considerable mismatches are tolerated, e.g., three non-consecutive mismatches [259]. Piwi proteins bind much larger small RNAs than Argonaute proteins (27–28 nt piRNAs versus 20–22 miRNAs) [160], and therefore may tolerate a higher number of mismatches at the 3′-end.

The difference in target interaction with miRNAs reflects the function of piRNAs as guardians against parasitic elements [7]. On one hand, targeting of piRNAs must occur by sufficiently stringent mechanisms such that silencing of cellular mRNAs is avoided. Control of the cleavage step is considered especially important because of the risk of entry into the ping-pong amplification cycle. On the other hand, tolerance of mismatches beyond the seed region constitutes a mechanism for adaptation to constantly evolving transposable elements [259].

The specificity of piRNA targeting was investigated in luciferase reporter assays that were designed as piRNA sensors. In mosquito Aag2 cells, the abundantly expressed tapiR1 could target 5′UTR, ORF and 3′UTR regions in transcripts, unlike most miRNAs that interact with 3′UTRs [140]. With respect to the seed region, one mismatch and three consecutive mismatches resulted in partial and complete de-silencing, respectively, and G:U wobble pairs were not tolerated. Interaction with the 3′ part of the piRNA was not absolutely required since silencing was observed with a target region that covers at least the 5′ half of the piRNA. As expected, the seed region was insufficient for silencing, indicating that more extensive interaction is needed [140,141]. Interestingly, mismatches at the predicted slicing site did not interfere with silencing. Similarly, in the silkworm, efficient triggering of the ping-pong signal was observed during the interaction of *Fem* piRNA with *Masc* mRNA in which there are three mismatches in the 3′ half. In the seed region, on the other hand, mismatches were not tolerated [177,182].

Besides the different rules for interaction with targets, miRNA and piRNA pathways differ considerably in other aspects. First, the diversity of piRNAs (millions of different sequences) in an organism is much greater than that of miRNAs (less than 1000) [63]. Because of this high diversity, it was predicted that almost any cellular mRNA potentially can be targeted by a piRNA, if allowing for three mismatches. However, while piRNAs are abundant in bulk, individual unique piRNA sequences are present at much lower levels and it can therefore be questioned if their cellular abundance is high enough to interact efficiently with targets and trigger silencing. When tested in *Drosophila* OSS cells using luciferase reporter assays, silencing efficiency was dependent on the abundance of piRNAs that targeted the transcripts. Moderate silencing of the reporter was only observed if the concentration of the targeting piRNAs was higher than 200 reads-per-million (rpm) while robust silencing required at least 2000 rpm [63]. By contrast, most miRNAs originate from introns of protein-encoding genes or are processed from independent transcripts [2]. Some miRNAs, as individual sequences, are expressed at very high levels in cells (to more than 100,000 rpm and 10,000 copies per cell; [258]) and it can be expected that their capacity for regulation of gene expression is very robust. Second, it needs to be taken into account that the silencing mechanism of miRNAs and piRNAs is very different. When Ago1 was artificially tethered to the 3′UTR of reporter transcripts, efficient silencing ensued, but this was not observed following tethering of Piwi (both in the absence of small RNAs) [63]. It is well documented that silencing by miRNAs results from the recruitment by Argonaute proteins of enzymes involved in mRNA degradation [261] while knowledge of the silencing mechanism of piRNAs includes the initiation of target cleavage and the activation of the ping-pong mechanism [262].

Because a high number of sequences of transposable elements are incorporated in piRNA clusters, the amounts of piRNAs that accumulate may be sufficient to engage with their targets efficiently and cause transposon silencing. For the adaptation of the piRNA pathway to the regulation of cellular genes, however, it may be necessary for individual piRNAs to reach a critical threshold of expression in the cell. In the female silkworm, *Fem* piRNA is produced from a large number of repeats located in the sex-determining region of the W chromosome such that it can be readily identified by sequencing [182]. In culicine mosquitoes, piRNA clusters are found to be associated with satellite repeats from which individual piRNAs are expressed at high levels in a strongly phased pattern [136]. Satellite-derived piRNAs can accumulate at high levels in the mosquito embryo and regulate the degradation of maternal transcripts at the zygotic activation stage [140]. Also in the *Drosophila* testis, piRNAs that are derived from the *Su(Ste)* and *petrel* loci located on the Y chromosome, are expressed at high levels and play essential roles during spermatocyte differentiation [11]. It was also noted that 3′UTRs of many cellular mRNAs contain sequences from transposable elements [10], pointing to an obvious mechanism by which the piRNA pathway can expand its function to cellular gene regulation [262].

In addition to the canonical piRNA pathway that is modeled on transposon silencing, Piwi proteins clearly can have more disparate functions such as the transport and localization of mRNAs and piRNA/mRNA complexes by Aub during oocyte differentiation in *Drosophila* [69,70]. In these instances, association with targeted mRNAs is considered much more relaxed and may sometimes not involve piRNAs [70]. Furthermore, silencing may not be carried out by slicing but by recruitment of mRNA degradation enzymes and interaction with the miRNA pathway [72,77]. More research is required to unravel the possible role of Aub-like proteins in the transport and subcellular localization of piRNAs and mRNAs in both gonadal and non-gonadal (e.g., neuronal) tissues of *Drosophila* and other insects.

In gonadal somatic cells of *Drosophila*, Piwi localizes in the nuclei to carry out the transcriptional repression of transposable elements which, however, does not require its slicer activity [263]. For the mapping of Piwi target sites at the chromatin, binding of wild-type Piwi was compared with binding of a Piwi mutant that is deficient in piRNA binding [233]. Although many piRNA-dependent target sites were identified, especially at the 5′ and 3′ ends of protein-encoding genes, interaction of mutant Piwi at many unique and overlapping sites was also detected, indicating that Piwi can be recruited to genomic sites by other mechanisms. This phenomenon was observed despite the severely decreased capacity of mutant Piwi proteins to localize in the nuclei in the absence of interaction with piRNAs. Thus, Piwi likely has functions in the nucleus that are independent of piRNAs, for instance through interactions with HP1 proteins [86].

### 6.3. Expansion of the Function of piRNAs: From Transposon Control to Gene Regulation and Antiviral Defense

The piRNA pathway is considered to have higher adaptability than the miRNA or siRNA pathways because piRNAs are, first, processed from single strand RNA, a substrate that is much more abundant in the cell than dsRNA, and secondly by a mechanism that is imprecise and intrinsically generates variety [28,255].

For the activation of the piRNA pathway, long piRNA precursor RNAs are transcribed in the nucleus and become recruited to nuage-like granules that have mitochondrial and perinuclear localization [264]. Nuage-like granules represent membraneless condensates that spontaneously assemble by multivalent protein-protein, RNA–RNA and protein–RNA interactions to enrich for functional components that compose specific biochemical pathways [265]. Essential for the formation of condensates is the phenomenon of liquid-liquid phase separation which drives the sorting of proteins and RNAs above a critical concentration. Transcripts from piRNA clusters may nucleate the formation of the granules [266] while constituent proteins possess motifs that enable multiple interactions, e.g., intrinsically disordered regions in Vasa proteins and Tudor domains in the eponymous Tudor proteins [267]. 

Species-specific expansion of the proteins in the piRNA pathway may be indicative for the formation of different types of nuage-like granules that carry out new functions of piRNAs. More specifically, Tudor proteins are implicated in the organization of condensates with specific functions: in mosquito cells, Ven-bodies and Attari-PB granules may represent distinct steps in piRNA biogenesis or function [109,111] (see Section 3.2). In the fly, Tudor proteins determine the assembly of nuage at the outer mitochondrial membrane (primary and phased piRNA production) and perinuclearly (ping-pong amplification) [28,268]. In addition, the Tudor proteins Kimper and Qin regulate recruitment of Aub and Ago3 to stimulate heterotypic ping-pong amplification for the preferential production of antisense transposon piRNAs [269]. Although the silkworm genome encodes only two Piwi genes, multiple types of membraneless granules related to the piRNA pathway can be observed in BmN4 cells that can be associated with separate biochemical processes (e.g., [162,166]; see Section 4.1).

The compartmentalization of the piRNA components in nuage-like granules could also play a role in the specific recruitment of piRNA precursors and the exclusion of other transcripts such as mRNAs and long non-coding RNAs (lncRNAs). In the nurse cells of the *Drosophila* ovary, export of piRNA precursor transcripts from the nucleus is directly coupled to the loading in the nuage granules [270]. Similarly, transcripts from the *flamenco* piRNA cluster possess discrete sequence elements that bind the Tudor protein Yb for localization to Yb bodies in somatic cells of the ovary [271]. However, it was demonstrated that in *Drosophila* cells that express few piRNA clusters, mRNAs and tRNAs were processed to piRNAs by the primary pathway [93], indicating the possibility for recognition by the piRNA machinery by a non-specific mechanism. During RNA virus infections, high levels of primary vpiRNAs are also often observed that correspond to the positive strand of the viral genomes [13,197]. This phenomenon may be explained by the high accumulation of viral RNA genomes and mRNAs in the cells that may lead to their non-specific recruitment by the piRNA machinery.

Non-specific incorporation of cellular mRNAs could also explain the existence of piRNAs derived from protein-coding genes, of which the function remains to be elucidated [10,16]. The expansion of the piRNA pathway into direct cellular gene regulation is nevertheless very convincingly demonstrated in several physiological processes that were already discussed, such as spermatogenesis in *Drosophila*, sex determination in *Bombyx* and maternal mRNA degradation in *Aedes*. In these well-documented cases, however, high levels of individual regulatory piRNAs are detected, which suggests that specialized mechanisms of production are necessary, e.g., the expansion of repeats in the genome and the phased processing of repeat piRNA precursors [136]. Similarly, it remains to be investigated whether a mechanism exists for the recruitment of the target mRNAs to specific nuage-like granules with unique composition.

The expansion of the Piwi genes in mosquitoes for the acquisition of a new functional role in antiviral defense comprises an interesting phenomenon. For instance, it can be asked whether the different Piwi proteins could localize to different types of nuage-like granules that represent unique functions [103]. More specifically, do specific condensates exist that are uniquely dedicated to antiviral immunity and how do they recruit vpiRNA precursor transcripts? Because Piwi4 was found to interact with Ago2 in *Ae. aegypti* [96], the formation of particular “hybrid” granules could be triggered that integrate the piRNA and siRNA pathways for antiviral defense. With respect to the activation of the pathway, it is noted that the formation of vDNAs seems to be necessary for a robust antiviral response by the ping-pong mechanism [96]. Further research is required about the function of vDNA forms: for instance, could they function as episomal piRNA clusters in the nucleus for the transcription of vpiRNA precursor transcripts (and therefore act identically to genomic piRNA clusters)? From this perspective, the formation of vDNA forms may be necessary as a mechanism for the efficient integration in the canonical piRNA pathway.

### 6.4. The piRNA Pathway as Guardian of the Genome: Implications for Stem Cell Function

Piwi genes were originally identified by their role in stem cell function in the gonads [51] and only later their major function in the defense against transposable elements was elucidated [7,9,28]. It is clear, however, that the integrity of the genome is much more important in germ cells than in somatic cells because of the potentially harmful impact on embryonic development of transposon activation. The preferential expression of Piwi proteins in gonadal tissues is therefore observed in many insect species belonging to different orders [168,214,251]. Similarly, stem cells can be expected to have higher levels of expression of the piRNA pathway because of the risk of adverse effects in the descendant cells that (re)generate functional tissues. In organisms with high regeneration capacity, such as planarians and the cnidarian *Hydra*, pluripotent stem cells are present that contain high levels of Piwi proteins [272,273,274]. In animals with more limited regeneration capacity, which include insects, the potential of stem cells is often restricted to the renewal of cell types within a single tissue [275]. In mammals, Piwi proteins were enriched in hematopoietic stem cells but not in epidermal or intestinal stem cells, questioning a general role for Piwi genes in adult stem cell function. The widespread expression of Piwi proteins in somatic tissues of insects (with the important exception of drosophilid dipterans) invites the question whether particular cell types can be enriched for Piwi protein expression, such as stem cells.

In *Drosophila*, Piwi protein expression plays a variable role in germline stem cell function [33,71]. As an example, the stem cell niche cells require Piwi expression for germline stem cell maintenance in the ovary but not in the testis [52,61]. From the (small amount of) information that is available (in a single insect species), no simple relationship seems to exist between Piwi expression and stem cell function during normal physiological and developmental conditions (discussed in Section 2.2).

In pathological conditions, however, the importance of expression of Piwi genes for stem cell maintenance may become more obvious. During pathogenic infection or after damage, expression of Piwi is induced in the regenerative stem cells and enteroblasts of the *Drosophila* midgut [276]. In Piwi mutants, the regenerative capacity of the midgut was diminished which led to decreased survival. The loss of Piwi was accompanied with an increase in transposon mobilization, which resulted in an increase in DNA damage and the induction of apoptosis in the gut stem cells [276]. Additionally, during aging, increased expression of transposons is observed in tissues, which is related to a decrease in Piwi function. This is not only observed in the midgut of the fly but also in the niche of the germline stem cells in the ovary [277]. The gradual loss of Piwi results in the activation of the Toll immune signaling pathway by mobilized retrotransposons and the deterioration of the mechanism of anchoring of the stem cells to the niche cells (which involves the degradation of β-catenin and the disruption of the *trans*-interaction between extracellular E-cadherin domains; [277]).

These examples from *Drosophila* show the role of Piwi proteins as important guardians of genome integrity during stress conditions and intense proliferation, which may also apply in other insects even if their Piwi factors (as Aub and Ago3 homologs) may preferentially locate in the cytoplasm. Other forms of genomic stress are encountered during cancer development and DNA virus replication in the nucleus. While in mammals the role of the piRNA pathway in the regulation of cancer cell function has received wide interest [278], its involvement in virus infection remains understudied [279], which could be related to the preferential expression of Piwi proteins in the germline [280]. Interestingly, the Piwi proteins Aub and Ago3 have recently been shown to have a proviral role during the infection of BmNPV (*Baculoviridae*) in silkworm larvae (discussed in Section 4.4; [204]). It can be speculated that the Piwi proteins have a role in the stabilization of transposon mobilization that could interfere with faithful viral genome replication. Changes in transposon expression were indeed documented during several viral infections that involved baculoviruses [207], flaviviruses [281], alfaviruses [130] and geminiviruses [223]. 

## 7. Conclusions

The piRNA pathway originally emerged as a defense mechanism against transposon mobilization in the genome, most critically in the germline cells. Its function requires the existence of transcription units known as piRNA clusters that locate at heterochromatin and repeat regions in the genome in which transposable elements accumulate. In antisense orientation, the piRNA cluster transcripts then operate as sensors for the expression of transposable elements which become silenced by the activation of the ping-pong loop. Expansion of the piRNA pathway to gene regulation (for instance, during spermatogenesis, sex determination and at the maternal/zygotic transition during early embryogenesis) has also been observed but this may have required the acquisition of a mechanism for the production of unique piRNAs at sufficiently high levels, for instance through phased piRNA accumulation at repetitive elements. Similarly, development of the antiviral defense function could have occurred by a mechanism in which viral RNA sequences became recognized as piRNA precursors, for instance through interaction with retrotransposon elements. In most insects (with drosophilid flies as major exception), piRNAs and Piwi proteins are also abundantly expressed in somatic tissues, with possible functions in tissue regeneration, aging, stem cell function and splicing. Finally, Piwi proteins have been associated with RNA transport and localization, such as during oocyte development.

## Figures and Tables

**Table 1:** Overview of physiological and developmental processes that are regulated by the piRNA pathway in different insects. Hypothetical functions are indicated with a question mark.

Species	Tissue	Process
*Drosophila melanogaster* (Diptera)	Ovary	Transposon control (transcriptional silencing): somatic cells Transposon control (posttranscriptional silencing): germline cells Stem cell function Telomere maintenance (germline cells) mRNA/piRNA transport and localization (nurse cell-oocyte complex)
	Testis	Stem cell function Spermatocyte differentiation Regulation of fertility
	Somatic tissues	Transposon control Stem cell function
	Embryo	Maternal mRNA degradation (early embryogenesis)
*Aedes aegypti* *Aedes albopictus* (Diptera)	Aag2 cells Somatic tissues	Transposon control (posttranscriptional silencing) Antiviral defense Immunological memory?
	Embryo	Maternal mRNA degradation (early embryogenesis)
*Bombyx mori* (Lepidoptera)	BmN4 cells	Transposon control (posttranscriptional silencing) Antiviral defense? Proviral role during baculovirus infection? Stress response?
	Embryo	Maternal mRNA degradation (early embryogenesis) Sex determination
*Blatella germanica* (Blattodea)	Embryo	Maternal mRNA degradation (early embryogenesis)?
*Macrotermes bellicosus* (Blattodea)	Whole body	Transposon control (related to caste differentiation)
*Locusta migratoria* (Orthoptera)	Somatic tissues	Transposon control Splicing regulation
*Bemisia tabaci* (Hemiptera)	Somatic tissues	Transposon control
*Diaphorina citri* (Hemiptera)	Somatic tissues	Antiviral defense?
*Acyrthosiphon pisum* (Hemiptera)	Ovary	Oocyte differentiation? Parthenogenetic embryogenesis?
*Rhodnius prolixus* (Hemiptera)	Ovary	Oocyte differentiation
*Tribolium castaneum* (Coleoptera)	Embryo	Maternal mRNA degradation (early embryogenesis)
*Apis mellifera* (Hymenoptera)	Whole body	Transposon control (related to caste differentiation)
	Embryo	Maternal mRNA degradation (early embryogenesis)?

## Data Availability

The analysis is based on published scientific literature which is publicly accessible.

## Supplementary Materials

The following supporting information can be downloaded at: https://www.mdpi.com/article/10.3390/insects14020187/s1. Supplementary Table S1 and Supplementary Table S1 legend.
